# Developmental mechanism of the periodic membrane skeleton in axons

**DOI:** 10.7554/eLife.04581

**Published:** 2014-12-23

**Authors:** Guisheng Zhong, Jiang He, Ruobo Zhou, Damaris Lorenzo, Hazen P Babcock, Vann Bennett, Xiaowei Zhuang

**Affiliations:** 1 Department of Chemistry and Chemical Biology, Howard Hughes Medical Institute, Harvard University, Cambridge, United States; 2 Department of Molecular and Cellular Biology, Howard Hughes Medical Institute, Harvard University, Cambridge, United States; 3 Department of Biochemistry, Duke University, Durham, United States; 4 Department of Neurobiology, Duke University, Durham, United States; 5 Center for Brain Sciences, Harvard University, Cambridge, United States; 6 Department of Biochemistry, Howard Hughes Medical Institute, Duke University, Durham, United States; 7 Department of Physics, Harvard University, Cambridge, United States; Albert Einstein College of Medicine, United States

**Keywords:** actin, spectrin, ankyrin, axon, super-resolution, STORM, mouse, rat

## Abstract

Actin, spectrin, and associated molecules form a periodic sub-membrane lattice structure in axons. How this membrane skeleton is developed and why it preferentially forms in axons are unknown. Here, we studied the developmental mechanism of this lattice structure. We found that this structure emerged early during axon development and propagated from proximal regions to distal ends of axons. Components of the axon initial segment were recruited to the lattice late during development. Formation of the lattice was regulated by the local concentration of βII spectrin, which is higher in axons than in dendrites. Increasing the dendritic concentration of βII spectrin by overexpression or by knocking out ankyrin B induced the formation of the periodic structure in dendrites, demonstrating that the spectrin concentration is a key determinant in the preferential development of this structure in axons and that ankyrin B is critical for the polarized distribution of βII spectrin in neurites.

**DOI:** http://dx.doi.org/10.7554/eLife.04581.001

## eLife digest

The brain contains hundred types of neurons, but they are all variations on the same basic structure. Each neuron consists of a cell body that is covered in short protrusions called dendrites and a long thin structure called the axon. The dendrites receive incoming signals from neighboring neurons and they transmit these signals via the cell body to the axon, which in turn relays them to the dendrites of the next neuron (or neurons).

Like all cells, neurons maintain their structure with the help of an internal cytoskeleton made up of many different proteins. However, it was discovered recently that axons have an additional lattice-like structure underneath their outer membrane. This structure, which consists of rings of actin filaments separated by molecules of a protein called spectrin, is preferentially formed in axons and is found much less frequently in dendrites.

Now Zhong, He et al., who are members of the research group that discovered the axonal skeleton, have used ‘super-resolution imaging’ to figure out how this skeleton forms and why it predominantly forms in axons. In brief, a basic version of the sub-membrane periodic skeleton is laid down early in development, starting next to the cell body before gradually spreading down the axon. The skeleton then continues to mature throughout development with the incorporation of several additional types of proteins.

The periodic skeleton only forms in regions which contain enough βII spectrin. Under normal conditions, dendrites contain too little βII spectrin to support the growth of such a periodic skeleton. However, artificially increasing the amount of βII spectrin present by overexpressing the corresponding gene, or by knocking out ankyrin B (a molecule that is important for establishing the preferential distribution of βII spectrin in axons), is sufficient to trigger periodic skeleton formation in dendrites. Given that axons and dendrites have distinct roles in neuronal signaling, this uneven distribution of spectrin is likely to be one way in which these regions maintain the specific structures that support their individual functions.

**DOI:** http://dx.doi.org/10.7554/eLife.04581.002

## Introduction

Neurons are highly polarized cells with their somatodendritic regions receiving synaptic inputs and axons propagating electrical signals and sending synaptic outputs to target cells. Cytoskeletal proteins are important for maintaining the polarity of neurons. For example, actin and microtubules are essential for the growth and stabilization of axons, the trafficking of cargos to specific neurites, and the stabilization and plasticity of synapses (Luo, 2002; Dent and Gertler, 2003; Cingolani and Goda, 2008; Barnes and Polleux, 2009; Kapitein and Hoogenraad, 2011; Stiess and Bradke, 2011). Transient destabilization of actin at the tip of a neurite is sufficient to induce a dendrite to become an axon (Bradke and Dotti, 1999). Increasing evidence also suggests an important role for spectrin in the maintenance of neuronal polarization, as well as the development and stabilization of axons (Hammarlund et al., 2007; Galiano et al., 2012). αII and βII spectrin are enriched in axons (Riederer et al., 1986; Galiano et al., 2012). Spectrin is known to be important for providing the mechanical stability for axons (Hammarlund et al., 2007) and protecting them from mechanical stress (Krieg et al., 2014), for axon path finding (Hulsmeier et al., 2007), for the stabilization of pre-synaptic terminals (Pielage et al., 2005), and for maintaining specific membrane domains in axons (Susuki and Rasband, 2008). Mice lacking either αII or βII spectrin die in the embryo, highlighting the crucial function of these proteins (Tang et al., 2003; Stankewich et al., 2011; Galiano et al., 2012). Spectrin has also been shown to play a role in human neurological diseases (Ikeda et al., 2006; Writzl et al., 2012).

Recently, we discovered a periodic sub-membrane lattice structure made of actin, spectrin, and other associated molecules in the axons of mammalian neurons (Xu et al., 2013). In this membrane skeleton, actin filaments form a ring-like structure that wraps around the circumference of axons. These actin rings are evenly spaced along the axon shaft with a period of ∼190 nm and show a remarkably long-range order. Actin filaments in the rings are capped by adducin. Adjacent actin rings are connected by spectrin, likely in the form of αII-βII-spectrin heterotetramers, given the observations that the periodic βII spectrin rings alternate with the actin–adducin rings along axons and that the observed 190 nm period matches the length of the spectrin tetramer. The ultrastructural organization of this quasi-one-dimensional, periodic lattice structure is different from the previously observed, two-dimensional polygonal membrane skeletal structure found in red blood cells (Byers and Branton, 1985; Liu et al., 1987; Bennett and Lorenzo, 2013), whereas an erythrocyte-like polygonal membrane skeletal was observed in the axon terminals at the *Drosophila* neuromuscular junction (Pielage et al., 2008). Interestingly, this periodic structure preferentially forms in axons, with the actin in dendrites primarily adopting the form of long filaments running along the dendrite shaft (Xu et al., 2013). In *Caenorhabditis elegans* that lack β spectrin or carry a β spectrin mutant, axons break more easily during animal movement (Hammarlund et al., 2007) and exhibit impaired touch sensation (Krieg et al., 2014), suggesting that this structure may be important for the mechanical stability of axons and for sensing mechanical stimuli. This periodic lattice also organizes the axonal membrane by placing important membrane proteins, such as the voltage-gated sodium channels, into a periodic distribution (Xu et al., 2013).

However, it is unknown how this highly regular membrane skeleton structure develops in axons and how its formation is regulated. For example, it is unclear whether the periodic lattice develops during early or late stages of axon differentiation. Although protein factors previously identified to be important for axon differentiation tend to be enriched and function at the growing tips of axons (Arimura and Kaibuchi, 2007; Barnes and Polleux, 2009; Stiess and Bradke, 2011; Cheng and Poo, 2012), it is unknown whether the actin–spectrin lattice also initiates at the distal ends of axons or instead forms first in the proximal region near the cell body. Finally, the molecular mechanism that regulates the specific formation of this periodic structure in axons, instead of dendrites, remains a mystery. In this study, we addressed these important questions concerning the development of this newly discovered neuronal structure. We found that the periodic membrane skeleton initiated early during axon differentiation. The lattice structure originated in the axonal region adjacent to the cell body and propagated to the distal ends of axons. The lattice structure further matured by recruiting other components, and the matured membrane skeleton was highly stable. Multiple molecular factors played roles in regulating the formation of this structure. The lattice structure depended on intact microtubules. The high local concentration of βII spectrin in axons was the key determining factor for the specific formation of the lattice structure in axons, and artificially increasing the concentration of βII spectrin in dendrites was sufficient to induce the formation of the periodic lattice structure in dendrites. Remarkably ankyrin B was important for the polarized distribution of βII spectrin in neurites; in ankyrin B knockout mice, βII spectrin was evenly distributed in axons and dendrites, giving rise to a highly regular, periodic membrane skeleton in both dendrites and axons.

## Results

### Early development and propagation of the periodic lattice structure in axons

Neurons exhibit distinct developmental stages with different morphological characteristics during polarization (Dotti et al., 1988; Arimura and Kaibuchi, 2007; Barnes and Polleux, 2009; Cheng and Poo, 2012). In dissociated hippocampal neuronal culture, neurons first display intense lamellipodial protrusive activity in stage 1, which then leads to the emergence of multiple immature neurites in stage 2 (∼1 Day in Vitro [DIV]). In stage 3 (DIV 2–4), one of these neurites breaks the symmetry and extends rapidly to become an axon. The other neurites then gradually acquire dendritic properties in stage 4 (DIV 4–7). In stage 5 (>DIV 7), neurons continue to mature and form axon initial segments, dendritic spines, and synapses. In order to determine the developmental course of the periodic membrane skeletal structure, we fixed dissociated neurons at different developmental stages, immunostained for βII spectrin, and imaged using stochastic optical reconstruction microscopy (STORM), a super-resolution imaging method that relies on switching and localizing single molecules to acquire sub-diffraction limit images (Betzig et al., 2006; Hess et al., 2006; Rust et al., 2006; Huang et al., 2008).

To illustrate how we systematically imaged and quantified this periodic structure in axons, we first imaged a neuron at DIV 10. Consistent with our previous findings (Xu et al., 2013), βII spectrin adopted a highly regular, periodic pattern in all regions of the axonal shaft (Figure 1—figure supplements 1, 2). Both Fourier transform and autocorrelation analyses showed that the βII spectrin adopted a periodic distribution with a period of ∼190 nm (Figure 1—figure supplement 1). Similarly, actin filaments exhibited a highly periodic distribution along axon shafts (Figure 1—figure supplement 3). Depolymerizing the actin filaments with latrunculin A (LatA) disrupted the periodic distribution of βII spectrin (Figure 1—figure supplement 2), and knocking-down βII spectrin using shRNA led to a loss of the periodic actin distribution (Figure 1—figure supplement 3). These results indicate that the periodic organizations of actin and spectrin are interdependent, consistent with the model that adjacent actin rings are connected by the spectrin tetramers.

**Figure 1.**
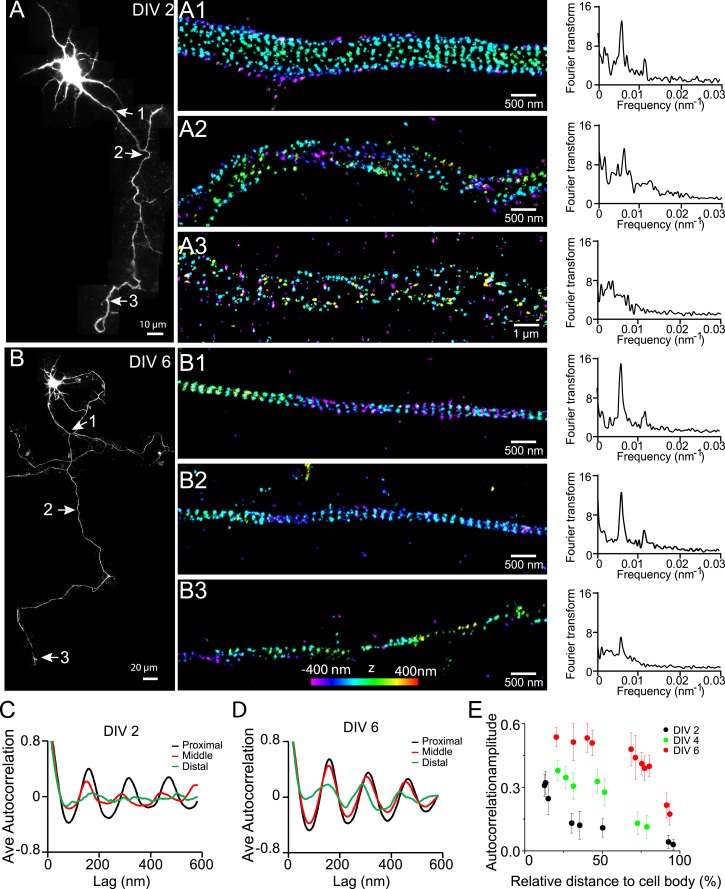
Early development and propagation of the periodic lattice structure in axons. (**A**) A DIV 2 neuron was stained with βII spectrin antibody and imaged by 3D STORM. The single long process from the cell is the axon. A1, A2, and A3 are 3D STORM images taken from arrow-indicated regions from **A**. The Fourier transform analyses of the βII spectrin distribution along the axon shaft are shown on the right. (**B**) Similar to (**A**), but for a DIV 6 neuron. (**C**–**D**) Autocorrelation analysis of βII spectrin distributions of DIV 2 (**C**) or DIV 6 (**D**) neurons at the proximal, middle, and distal regions of axons. Shown are the averaged autocorrelation from multiple segments of axons for each condition. (**E**) The average amplitude of autocorrelation analysis for different axonal regions of DIV 2, 4, and 6 neurons. The amplitude was measured as the difference between the first peak and the average of the two first valleys of the autocorrelation curve. Error bars are standard deviation from measurements of multiple neurons (n = 7 neurons for DIV 2; n = 6 neurons for DIV 4; n = 9 neurons for DIV 6; from three independent experiments at each DIV). The color bar for 3D STORM image indicates the z-depth of the image and is the same for all of our STORM images. **DOI:** http://dx.doi.org/10.7554/eLife.04581.003

Next, we quantified the distribution of βII spectrin at earlier developmental stages in DIV 2, 4, and 6 neurons (Figure 1). Figure 1A shows a typical stage 3 neuron at DIV 2, with one neurite outgrowing the others and becoming an axon. Interestingly, the periodic pattern of βII spectrin has already formed in the proximal region of this axon near the cell body, as shown by both Fourier transform and autocorrelation analyses of the STORM image (Figure 1A,C). However, the periodic distribution did not extend far—the middle and distal parts of the same axon did not exhibit the periodic pattern (Figure 1A,C). Similar results were observed for other stage 3 neurons that we imaged. As neurons continued to mature, the periodic βII spectrin distribution extended to more distal regions of axons. By DIV 6, the periodic βII spectrin distribution extended for nearly the entire length of the axon, except for the very distal region (Figure 1B,D). Using the autocorrelation amplitude at the first peak (∼190 nm) to quantify the degree of periodicity for the βII spectrin distributions, we found that the periodicity degraded quickly along axons in DIV 2 neurons but gradually extended to the distal end of the axon in later developmental stages until the structure eventually occupied nearly the entire axon (Figure 1E and Figure 1—figure supplement 4). Taken together, these results demonstrate that the periodic membrane skeleton forms early during development, originates in proximal axon regions close to the cell body, and propagates toward the distal end of the axon.

**Figure 1—figure supplement 1.**
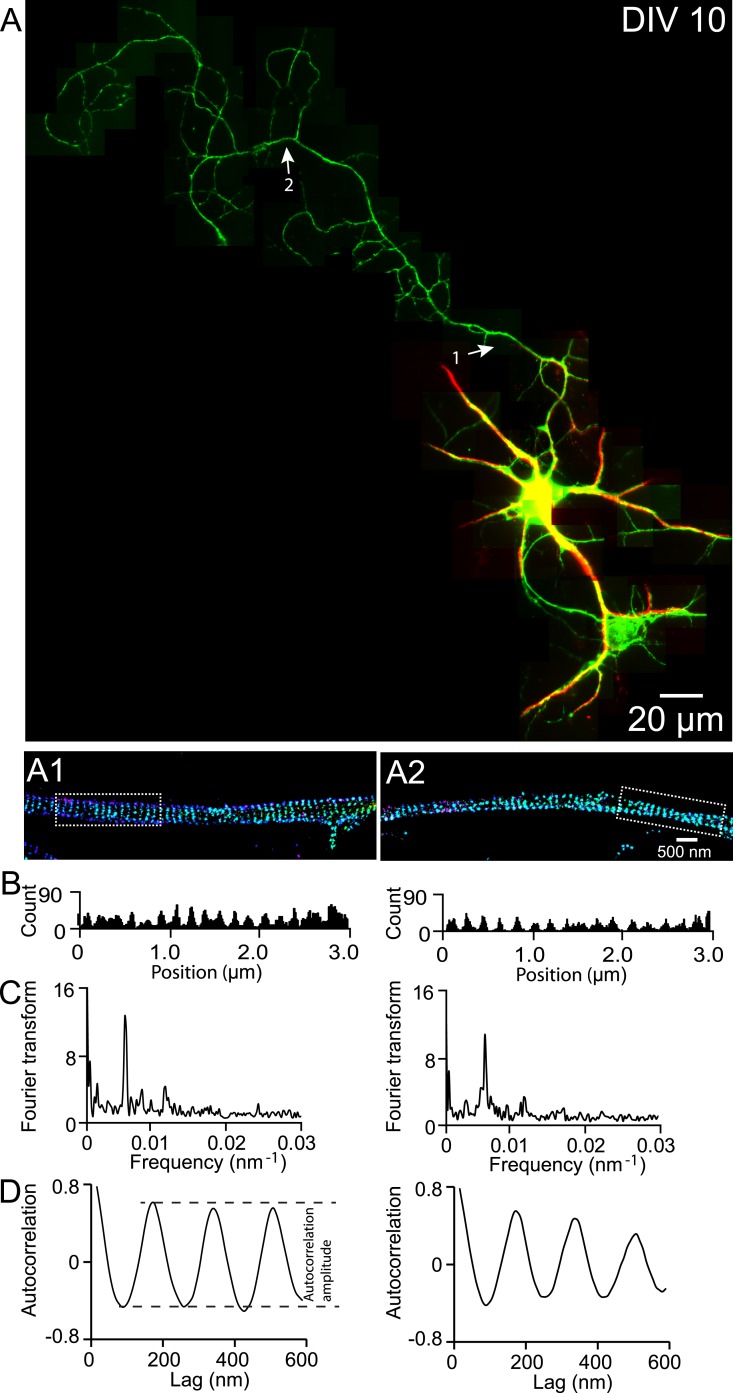
βII spectrin structure in a DIV 10 neuron. (**A**) A DIV 10 neuron was immunostained for βII spectrin (green) and Map2 (red) and imaged by conventional fluorescence and 3D STORM microscopy. STORM images of the two arrow-indicated regions along the axon are shown below. (**B**) Histograms of the βII spectrin localizations from the boxed regions in STORM images. (**C**) Fourier transform analyses of the βII spectrin localizations from the boxed regions. (**D**) Autocorrelation analyses of the βII spectrin localizations from the boxed regions. **DOI:** http://dx.doi.org/10.7554/eLife.04581.004

**Figure 1—figure supplement 2.**
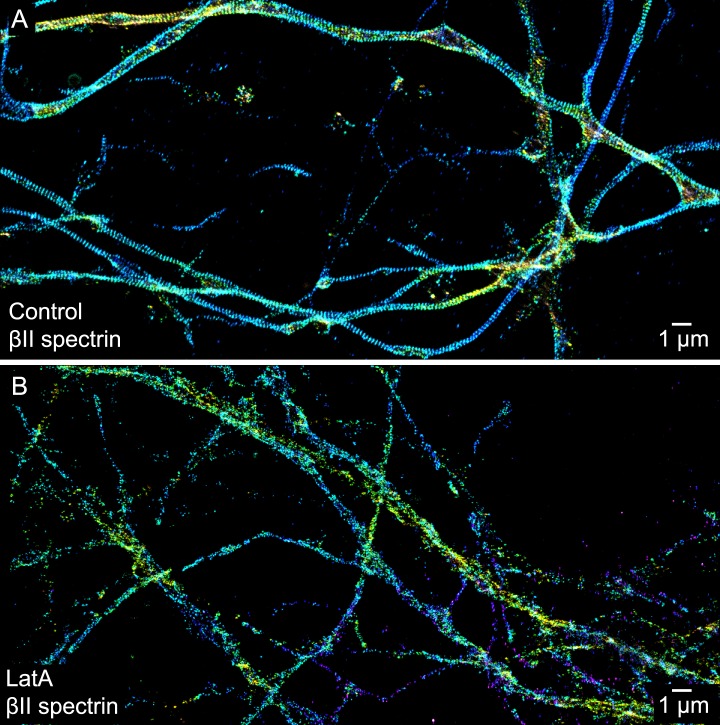
The periodic distribution of βII spectrin depends on actin. (**A**) STORM image of βII spectrin in axons of control DIV 10 neurons. (**B**) STORM image of βII spectrin of LatA-treated DIV 10 neurons (20 µM LatA, 1 hr). Similar results were observed in at least seven independent experiments. **DOI:** http://dx.doi.org/10.7554/eLife.04581.005

**Figure 1—figure supplement 3.**
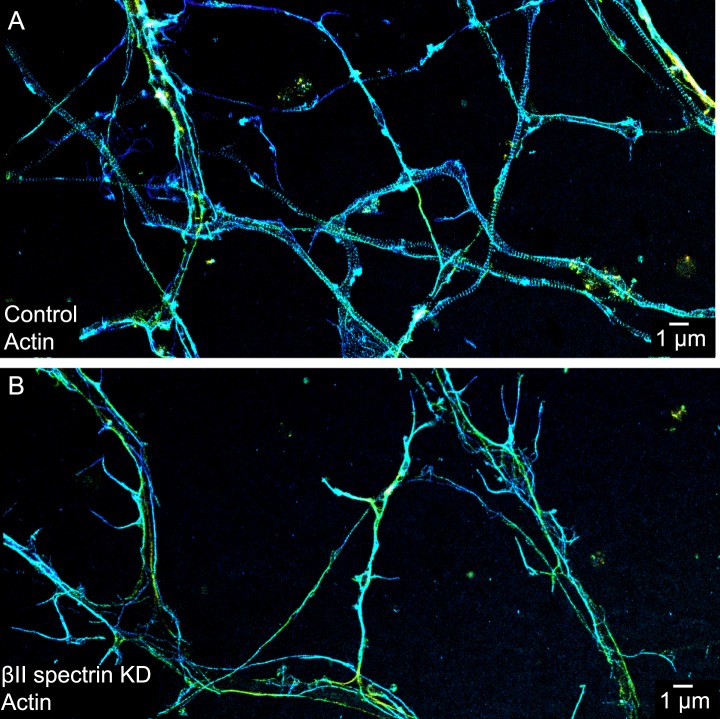
The periodic distribution of actin depends on βII spectrin. (**A**) STORM image of actin in axons of control DIV 10 neurons. In some of the thinnest axons, the presence of long actin filaments running along the axons can disguise the periodic structure due to finite resolution. (**B**) STORM image of actin in axons of βII spectrin-shRNA expressing neurons. Though we picked axon-enriched sample regions for imaging, we cannot preclude the possibility that a small number of the processes may be dendrites. Similar results were observed in 8 independent experiments. **DOI:** http://dx.doi.org/10.7554/eLife.04581.006

**Figure 1—figure supplement 4.**
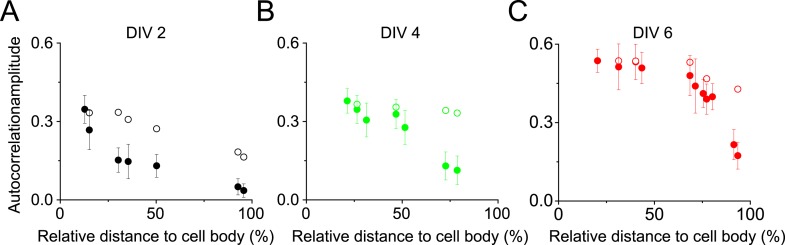
The autocorrelation analysis is not sensitive to the number of localizations present in the periodic structure within the range of the localization numbers that we detected in axon segments. Since the localization density of βII spectrin decreased towards the distal end of the axons, we tested whether the autocorrelation amplitude measured in Figure 1E would be sensitive to the localization numbers detected in various axon segments. (**A**) Filled circles: the autocorrelation amplitudes for different axonal regions of DIV 2 neurons reproduced from Figure 1E; open circles: the estimated dependence of the autocorrelation amplitude on the detected localization numbers if the periodic structure remained the same as that in the proximal axon region. To obtain these data, we randomly removed localizations from the proximal axon region to match the localization numbers measured in distal axons at various distances to the cell body, calculated the corresponding autocorrelation amplitudes, and plotted them here as the open circles. (**B**–**C**) Same as (**A**) except that the analyses were for DIV 4 and DIV 6 neurons. Note that the autocorrelation amplitude only decreases slightly when the localization number of a periodic structure was artificially reduced to match those measured in distal axon ends (open circles), and this slight change does not contribute substantially to the measured dependence of the autocorrelation amplitude on the distance to cell body (filled circles). **DOI:** http://dx.doi.org/10.7554/eLife.04581.007

**Figure 1—figure supplement 5.**
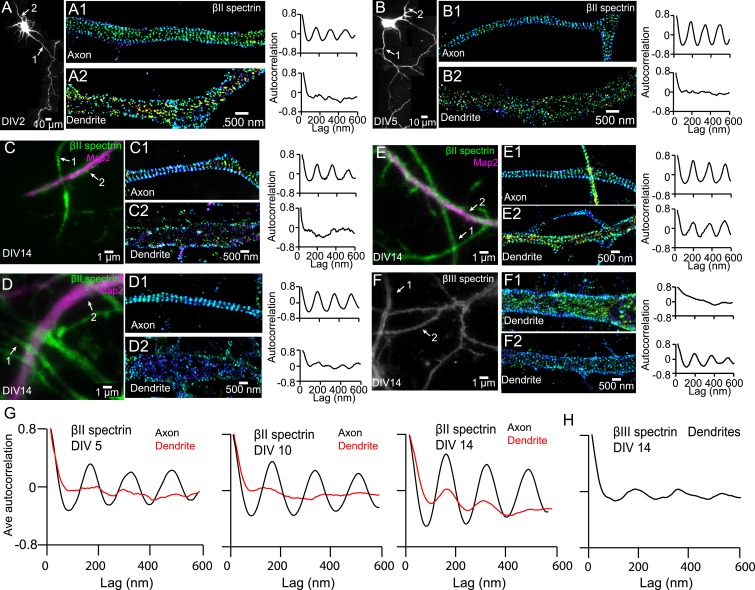
Distributions of dendritic βII and βIII spectrin. (**A**–**B**) DIV 2 and DIV 5 neurons were immunostained with βII spectrin, and both axonal and dendritic processes were imaged by 3D STORM. The reconstructed neuron image, 3D STORM images of axon and dendrite from indicated regions (arrows), and autocorrelation analyses are shown. (**C**–**E**) DIV 14 neurons were immunostained with βII spectrin and a dendritic marker Map2, and the βII spectrin was imaged by 3D STORM. The conventional images, 3D STORM images of axons and dendrites from indicated regions (arrows), and autocorrelation analyses are shown. (**F**) DIV 14 neurons were immunostained with βIII spectrin. The conventional image, STORM images of βIII spectrin from two indicated dendritic regions, and autocorrelation analyses are shown. βIII spectrin only stains for dendrites, and we did not observe detectable βIII spectrin in axons. (**G**) Average autocorrelation analyses of βII spectrin in axons and dendrites of neurons at DIV 5, DIV 10, and DIV 14 (n = 14 neurons for DIV 5; n = 11 neurons for DIV 10; n = 16 neurons for DIV 14; at least three independent experiments at each DIV). (**H**) Average autocorrelation analysis of βIII spectrin of DIV 14 neurons (n = 12 neurons, four independent experiments). **DOI:** http://dx.doi.org/10.7554/eLife.04581.008

**Figure 1—figure supplement 6.**
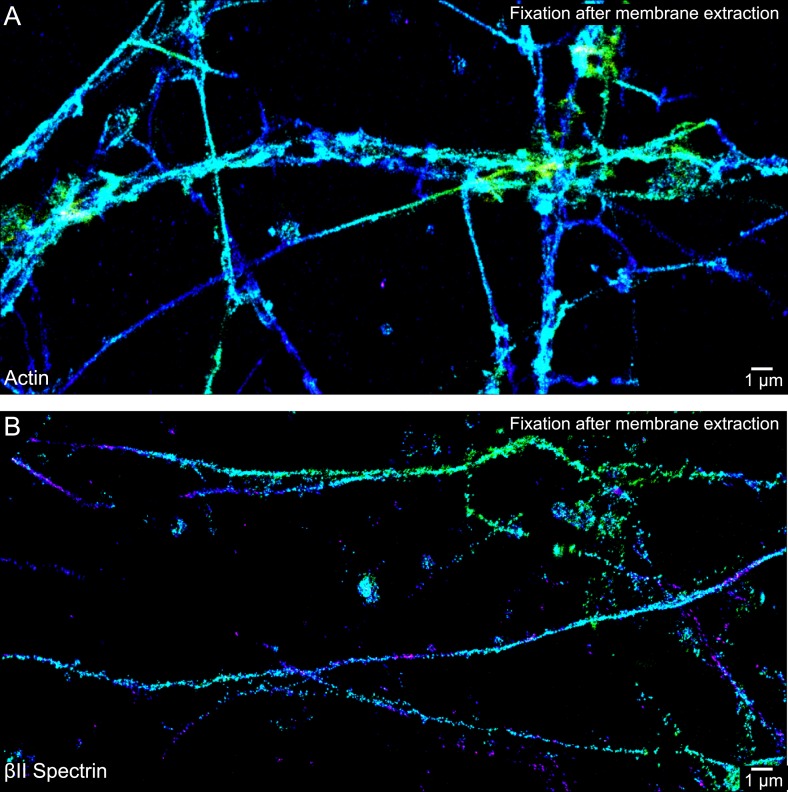
The periodic actin structure is disrupted if the neurons are subjected to membrane extraction prior to fixation. DIV 10 neurons were extracted with 1% Triton X-100 for 3 min, followed by two quick rinses, and then fixed with 0.2% GA for 20 min. See ‘Materials and methods’, ‘Fluorescence labeling of neurons’ section, for detailed procedure. 3D STORM images of actin and βII spectrin are shown in (**A**) and (**B**), respectively. Similar observations were found in six independent experiments. **DOI:** http://dx.doi.org/10.7554/eLife.04581.009

The periodic structure was only observed in axons, but not in dendrites, during early developmental stages, whereas isolated patches of periodic βII spectrin patterns were observed in dendrites during later developmental stages (Figure 1—figure supplement 5A–E). However, unlike in axons, these patches did not form a cohesive lattice structure with a long-range order. Quantitatively, the average autocorrelation analysis showed much smaller amplitudes in dendrites than those in axons (Figure 1—figure supplement 5G), indicating a much poorer regularity of the structure in dendrites. Similar results were observed for βIII spectrin (Figure 1—figure supplement 5F,H), an isoform of β spectrin that is enriched in dendrites instead of axons (Sakaguchi et al., 1998; Stankewich et al., 1998; Gao et al., 2011).

### Actin dependence during the early developmental phase of the periodic membrane skeleton

**Figure 2.**
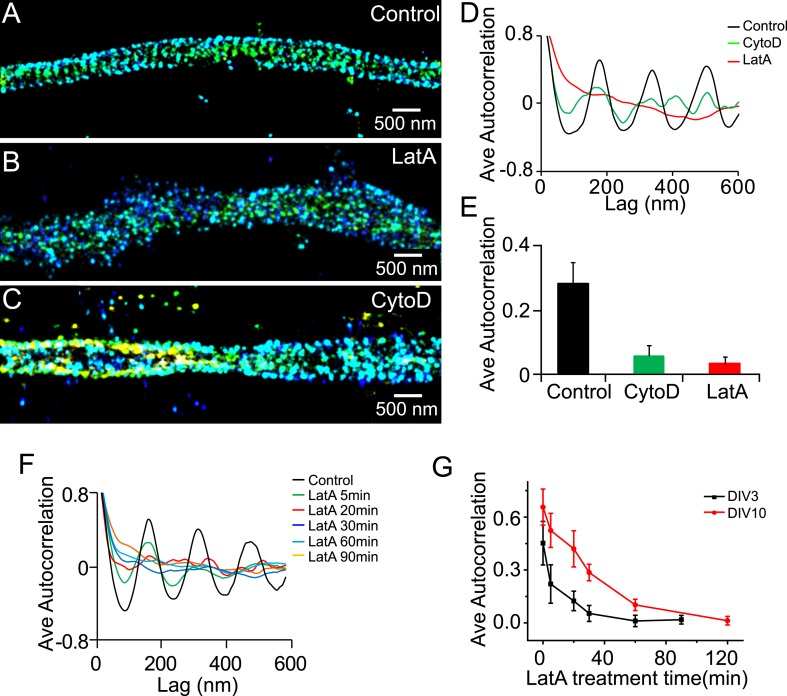
The periodic structure of βII spectrin depends on actin during early development. (**A**–**C**) DIV 3 neurons were either untreated or treated with latrunculin A (LatA, 20 µM) or cytochalasin D (CytoD, 50 µM) for 1 hr and subsequently immunostained with βII spectrin antibody for 3D STORM imaging. Shown here are representative images of βII spectrin in proximal axonal regions from control (**A**), LatA-treated (**B**) and CytoD-treated (**C**) neurons. (**D**) Average autocorrelation analyses of βII spectrin from multiple axon segments of control, LatA-treated, and CytoD-treated DIV 3 neurons (n = 6 neurons for control; n = 7 neurons for LatA-treated; n = 7 neurons for CytoD-treated conditions; at least three independent experiments for each condition). (**E**) The average autocorrelation amplitudes from control, LatA-treated, and CytoD-treated DIV 3 neurons. (**F**) Average autocorrelation analyses of βII spectrin from multiple axon segments of control and LatA-treated DIV 3 neurons at different treatment time (n > 5 neurons for each condition, three independent experiments). The axon segments are taken from the proximal axonal regions near the cell bodies. (**G**) DIV 3 and DIV 10 neurons were treated with 20 µM LatA for indicated amount of time, and the average autocorrelation amplitude of βII spectrin from these neurons are shown. Error bars are standard deviation from measurements of multiple neurons (n = 6 neurons for DIV 3; n = 8 neurons for DIV 10; four independent experiments at each DIV). **DOI:** http://dx.doi.org/10.7554/eLife.04581.010

Similar to the lattice structure in mature axons, the periodic pattern of βII spectrin depended on actin during early development. Treatment of neurons with actin-depolymerizing drugs, cytochalasin D (CytoD), or LatA disrupted the periodicity of βII spectrin in DIV 3 neurons (Figure 2A–E). The effect of actin-depolymerizing drugs set in quickly with the periodic βII spectrin distribution substantially disrupted after several minutes of LatA treatment (Figure 2F,G), consistent with the drug acting directly on the lattice structure. These results indicate that actin is involved in the lattice structure during early neuronal development. The form of actin, however, appeared to be different during the early developmental stages as compared to that in mature axons. We have previously shown that the periodic pattern of actin was not directly observed in the STORM images during DIV 1–4. In DIV 5, the periodic actin pattern begins to appear in some neurons and become robustly observed in neurons at DIV 7 (Xu et al., 2013). Similar results were observed here (data not shown). One possible interpretation is that actin existed in a less stable form during the early developmental stages and was not preserved by our sample treatment (fixation and extraction) prior to imaging. Consistent with the notion that actin filaments in the lattice structure were less stable during early developmental stages, the periodic structure of βII spectrin was more quickly disrupted by LatA treatment during early development than in older neurons (Figure 2G).

**Figure 3.**
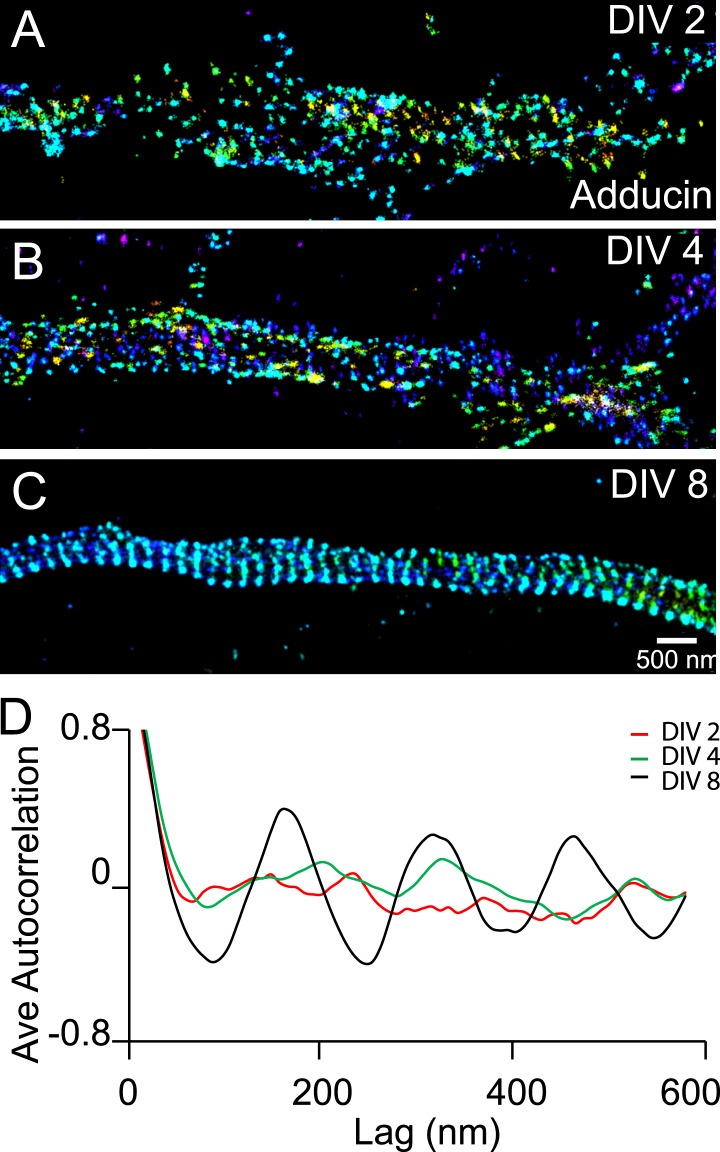
Recruitment of adducin into the periodic lattice structure during development. (**A**–**C**) Neurons were immunostained for adducin and imaged by 3D STORM. Shown here are representative images of adducin at proximal axonal regions from DIV 2, 4, and 8 neurons. (**D**) Average autocorrelation analyses of adducin at proximal regions of axons near the cell body. The autocorrelation curves are averaged from multiple neurons (n = 5 neurons for DIV 2; n = 7 neurons for DIV 4; n = 6 neurons for DIV 8; three independent experiments at each DIV). **DOI:** http://dx.doi.org/10.7554/eLife.04581.011

We also observed a relatively slow developmental time course for the periodic pattern of adducin, an actin-capping protein (Kuhlman et al., 1996). The periodic pattern of adducin was not observed in axons at DIV 2 or DIV 4 (Figure 3). A periodic pattern began to appear at ∼DIV 6 and became obvious after DIV 7 (Figure 3 and Figure 3—figure supplement 1). The lack of adducin capping may have contributed to the lower stability of actin during early development stages, although it is also possible that the lower stability of actin during the early development stages made it difficult to maintain the adducin pattern during cell fixation and extraction. Finally, it is formally possible that actin and adducin are not present in the periodic lattice structure during early developmental stages. However, we consider such a scenario to be less likely as it is difficult to imagine how spectrin tetramers themselves could self-assemble into a periodic lattice structure without the help of actin to crosslink multiple spectrin tetramers.

**Figure 3—figure supplement 1.**
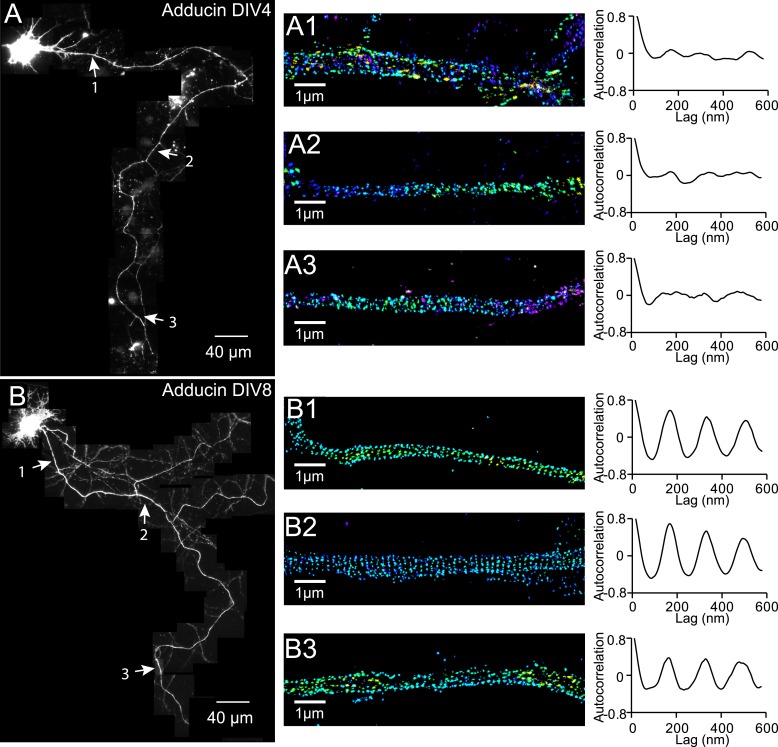
Distribution of adducin in axons. Neurons were immunostained for adducin and imaged by 3D STORM. The reconstructed neuron image, 3D STORM images of indicated regions (arrows), and corresponding autocorrelation analyses of DIV 4 (**A**) and DIV 8 (**B**) neurons are shown. **DOI:** http://dx.doi.org/10.7554/eLife.04581.012

Together, the above results indicate that the periodic membrane skeleton continued to mature after formation. Consistent with this notion, the autocorrelation amplitudes of the periodic βII spectrin distribution also continued to increase with time as the neuron matured (Figure 1E).

### Assembly of axon initial segment components into the periodic membrane skeleton during late development stages

As neurons further mature, axon initial segment (AIS) starts to assemble at the axonal region proximal to the cell body. Ankyrin G is the master regulating protein for AIS assembly and recruits other molecular components such as βIV spectrin and sodium channels to the AIS (Zhou et al., 1998; Jenkins and Bennett, 2001; Yang et al., 2007). Next, we examined whether βIV spectrin and ankyrin G were also recruited to the periodic membrane skeleton and, if so, during which developmental stage these components were incorporated.

**Figure 4.**
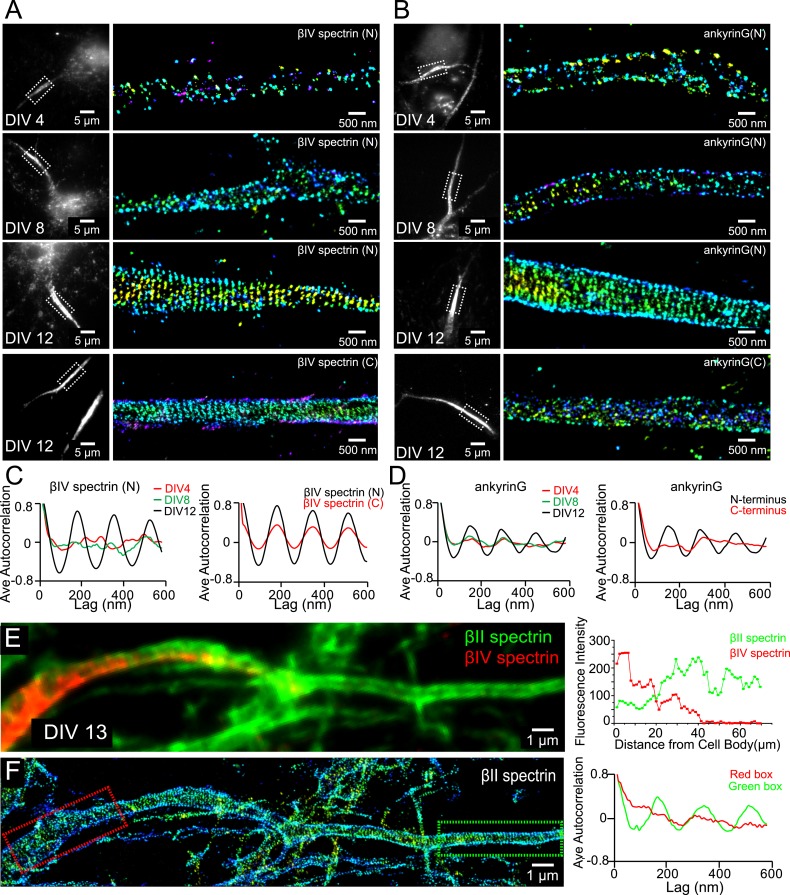
Assembly of AIS components into the periodic lattice structure during late developmental stages. (**A** and **B**) Neurons were immunostained with antibodies against βIV spectrin N-terminus (βIV spectrin (N)), βIV spectrin C-terminus (βIV spectrin (C)), ankyrin G spectrin-binding domain (ankyrin G (N)), or ankyrin G C-terminus (ankyrin G (C)), and imaged at various DIVs by 3D STORM. Representative conventional images and STORM images from the boxed region at different developmental stages are shown. (**C**) Left: average autocorrelation analyses of βIV spectrin N-terminus from neurons at different developmental stages (n = 9 neurons for DIV 4; n = 12 neurons for DIV 8; n = 16 neurons for DIV 12; at least three independent experiments at each DIV). Right: average autocorrelation analyses of βIV spectrin N-terminus and C-terminus of DIV 12 neurons (n = 13 neurons for N-terminus and n = 15 neurons for C-terminus, three independent experiments). (**D**) Left: average autocorrelation analyses of ankyrin G spectrin-binding domain (near N-terminus) from neurons at different developmental stages (n = 12 neurons for DIV 4; n = 15 neurons for DIV 8; n = 14 neurons for DIV 12; at least three independent experiments at each DIV). Right: average autocorrelation analysis of ankyrin G N-terminus and C-terminus of DIV 12 neurons (n = 9 neurons for N-terminus and n = 10 neurons for C-terminus, three independent experiments). (**E** and **F**) A DIV 13 neuron was immunostained with βIV and βII spectrin antibodies. βII spectrin was subjected for 3D STORM imaging. (**E**) Left: conventional image of βII and βIV spectrin in axon. Right: fluorescent intensity profile of βII and βIV spectrin along the axon. (**F**) Left: STORM image of βII spectrin in the same region. Right: autocorrelation analyses of βII spectrin from red- and green-boxed regions. **DOI:** http://dx.doi.org/10.7554/eLife.04581.013

We labeled ankyrin G using an antibody against its spectrin-binding domain near the N-terminus and βIV spectrin using an antibody against its N-terminal domain. Both ankyrin G and βIV spectrin signals were weak during early developmental stages, and the signals became stronger in the proximal region of axons at DIV 8 (Figure 4—figure supplement 1). Notably, the expression level of βII spectrin remained high throughout the axons during this time (Figure 4—figure supplement 1). At this time, the distributions of ankyrin G and βIV spectrin were not periodic (Figure 4A–D), in contrast to the highly periodic βII spectrin in the proximal region of axons (Figure 1). Over time, both ankyrin G and βIV spectrin signals further increased in the proximal region of axons, and by DIV 12, the N-terminal domains of both ankyrin G and βIV spectrin adopted highly periodic distributions, indicating that these molecules were incorporated into the periodic lattice structure (Figure 4A–D). Interestingly, the periodicity was substantially less pronounced for the C-terminal domain of βIV spectrin and undetectable for the C-terminal domain of ankyrin G (Figure 4A–D). These results suggest that the N-terminal regions of these molecules were tightly incorporated in the periodic lattice structure, but their C-terminal regions were likely hanging off from the lattice structure and moving relatively freely. Notably, as ankyrin G and βIV spectrin became incorporated into the periodic lattice, we observed a decrease in the local concentration of βII spectrin at the AIS (Figure 4E and Figure 4—figure supplement 1C). The decrease of the βII spectrin concentration was associated with a loss of periodicity for βII spectrin at AIS (Figure 4F), suggesting that as βIV spectrin was incorporated into the periodic structure in the AIS region, βII spectrin was displaced.

**Figure 4—figure supplement 1.**
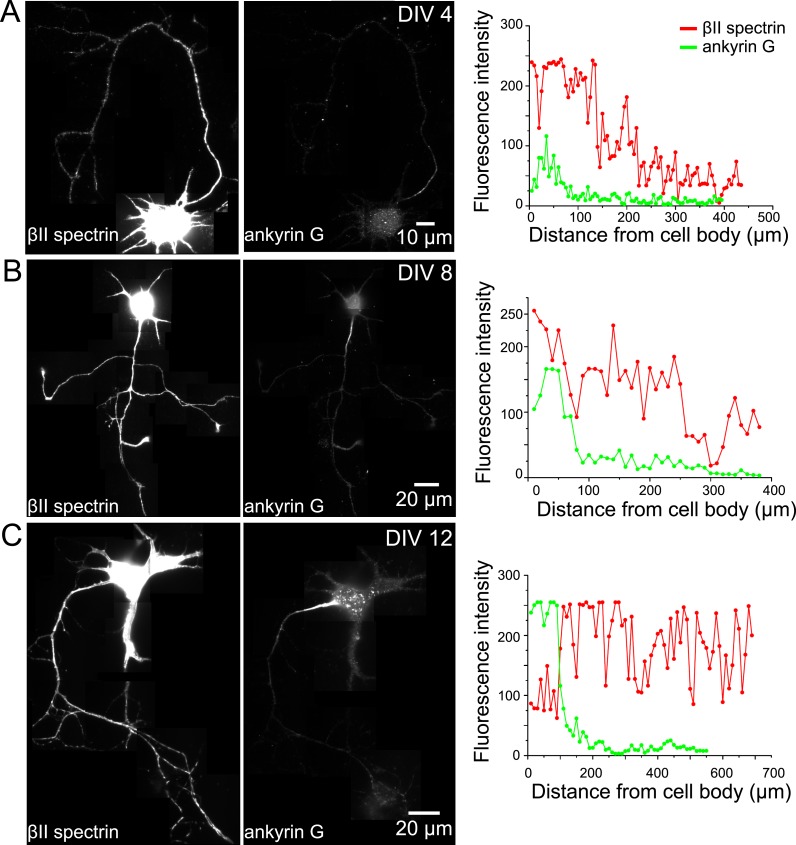
The expression profile of βII spectrin and ankyrin G in axons at different developmental stages. Neurons at DIV 4 (**A**), DIV 8 (**B**), and DIV 12 (**C**) were immunostained for βII spectrin and ankyrin G. Reconstructed neuron images based on βII spectrin fluorescence, ankyrin G fluorescence, and the fluorescence intensity profiles of both βII spectrin and ankyrin G along axons are shown. **DOI:** http://dx.doi.org/10.7554/eLife.04581.014

**Figure 4—figure supplement 2.**
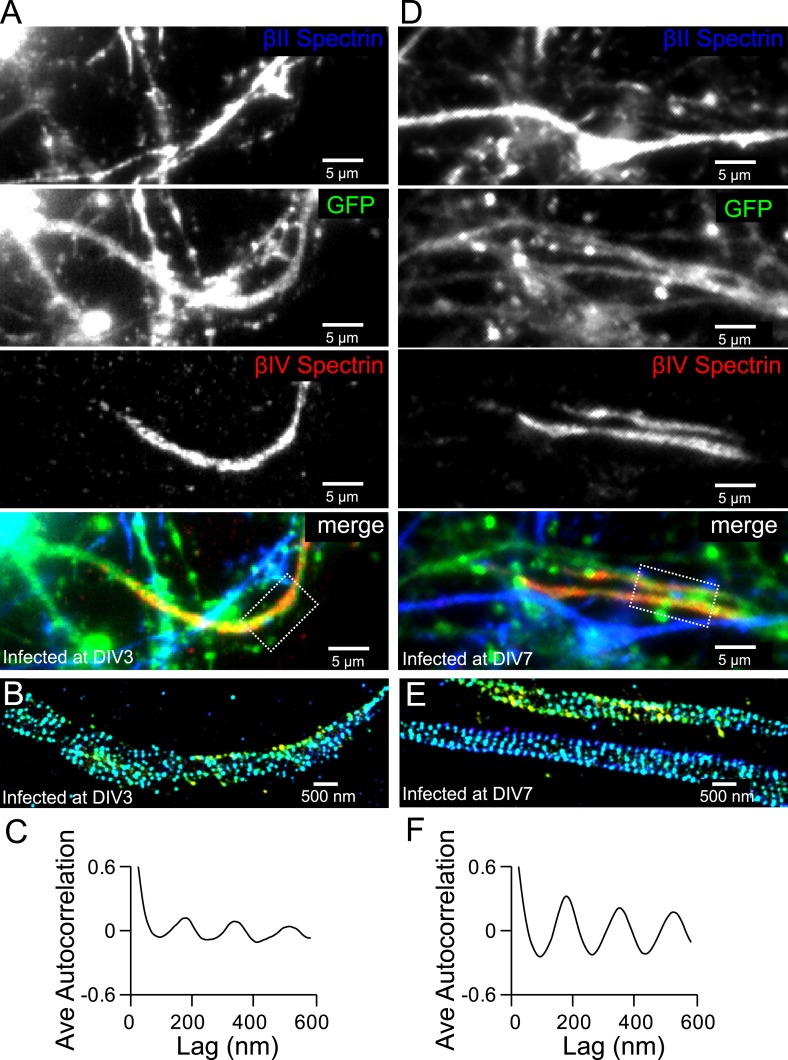
The formation of the periodic βIV spectrin structure in the AIS is dependent on βII spectrin. (**A**) Neurons were infected with βII spectrin-shRNA expressing adenovirus at DIV 3 and subsequently stained for βII spectrin and βIV spectrin at DIV 12. βIV spectrin was subjected for STORM imaging. Infected neurons have a GFP signal. The conventional images of βII spectrin, GFP, and βIV spectrin and the overlay image are shown. Efficient knockdown is demonstrated by lack of βII spectrin in GFP-positive process. (**B**) STORM image of βIV spectrin from boxed region in (**A**). (**C**) Average autocorrelation analysis of βIV spectrin from βII spectrin knockdown neurons (n = 11 neurons, three independent experiments). (**D**–**F**) Similar to (**A**–**C**) except that the virus was added at DIV 7 (n = 9 neurons, four independent experiments). **DOI:** http://dx.doi.org/10.7554/eLife.04581.015

To test whether the assembly of βIV spectrin into the periodic structure may rely on βII spectrin, we knocked down βII spectrin at various DIVs using a shRNA-expressing adenovirus, which also expressed GFP, and subsequently imaged βIV spectrin at DIV 12. The efficiency of knockdown was demonstrated by a lack of βII spectrin signal in virus-infected, GFP-positive neurons (Figure 4—figure supplement 2). When infected by the virus at DIV 3, the enrichment of βIV spectrin in the AIS region appeared partially impaired by βII spectrin knockdown, though at least 60% of the neurons still exhibited enrichment of βIV spectrin in AIS. For these neurons, the periodicity of βIV spectrin was also partially disrupted in the βII spectrin-depleted neurons (Figure 4—figure supplement 2A–C), indicating that βII spectrin is important for the periodic assembly of βIV spectrin. On the other hand, when neurons were infected with the virus at DIV 7, βIV spectrin remained periodic even though βII spectrin was depleted (Figure 4—figure supplement 2D–F). Because it takes several days for pre-existing βII spectrin molecules to degrade (Susuki et al., 2011), it is likely that βIV spectrin was already incorporated into the periodic lattice before the eventual depletion of βII spectrin when the virus was added late.

### Stability of the periodic membrane skeleton

**Figure 5.**
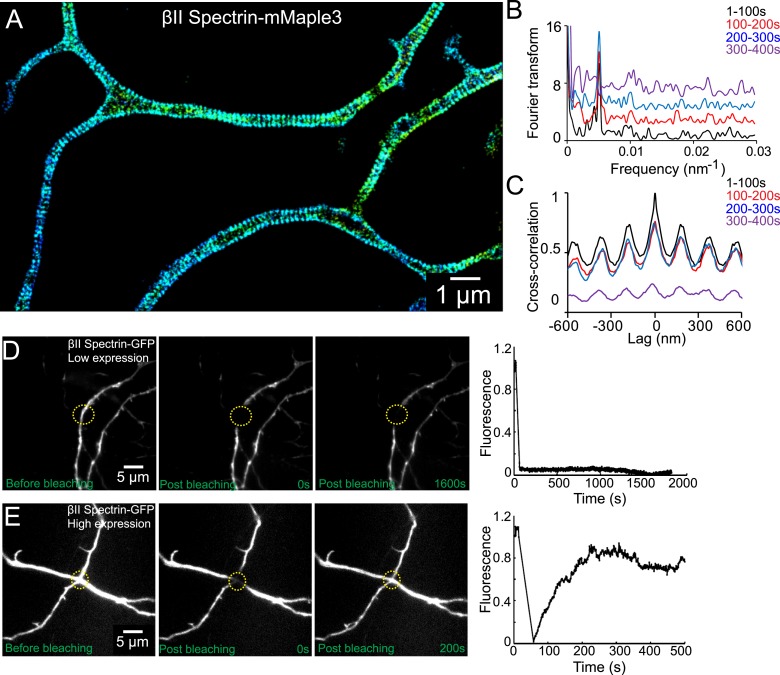
The periodic lattice structure is stable in live neurons. (**A**) 3D STORM image of βII spectrin-mMaple3 in live neurons at DIV 10. (**B**) The STORM movie was segregated into four different time windows. Fourier transform analysis of each time window is shown. The baseline of Fourier traces is shifted manually for clear visualization. (**C**) Cross-correlation analysis of βII spectrin across different time windows. The black curve is the autocorrelation of the image during 0–100 s. The color curves are the cross-correlation between 0–100 s and later time windows. Similar results were found in six independent experiments. (**D**–**E**) FRAP analyses of βII spectrin in DIV 10 neurons. Neurons were transfected with βII spectrin-GFP at DIV 8. (**D**) Representative neurons at a low βII spectrin expression level, where βII spectrin-GFP molecules were incorporated into the periodic structure. The images before photo-bleaching, 0 s post-bleaching, 1600 s post-bleaching, and the fluorescence recovery trace are shown. (**E**) The fluorescence recovery of representative neurons with a high βII spectrin expression level, where most βII spectrin-GFP molecules were not incorporated into the periodic structure. Similar results were found in at least nine independent experiments. **DOI:** http://dx.doi.org/10.7554/eLife.04581.016

We next probed the dynamics of the periodic lattice structure in live neurons. To this end, we genetically fused βII spectrin with mMaple3, a recently developed photoactivatable fluorescent protein (Wang et al., 2014). In neurons moderately expressing βII spectrin-mMaple3, the periodic pattern of βII spectrin-mMaple3 was readily observable in axons and the spacing of ∼190 nm was identical to that observed for endogenous βII spectrin in fixed neurons (Figure 5A). The periodic pattern was smeared in neurons with high expression levels of βII spectrin-mMaple3, presumably by the excess, freely diffusing βII spectrin-mMaple3 molecules that were not incorporated into the lattice structure.

**Figure 5—figure supplement 1.**
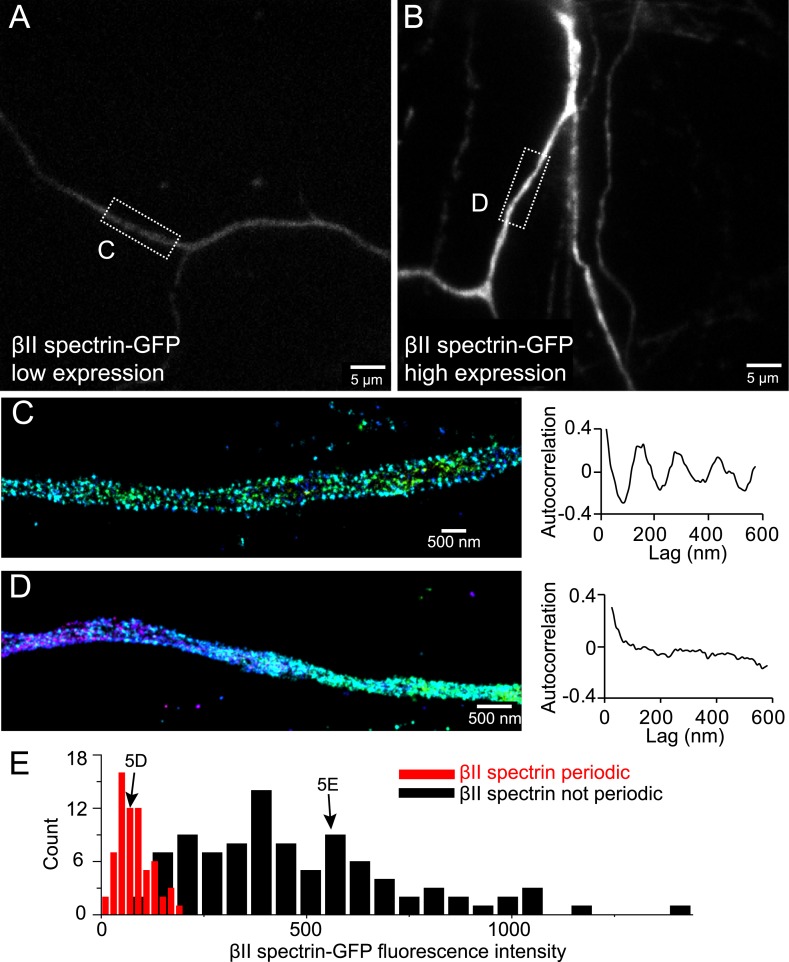
Incorporation of βII spectrin-GFP into the periodic lattice structure in overexpressing neurons. Neurons were transfected with βII spectrin-GFP at DIV 8 and immunostained for GFP at DIV 10. Representative conventional images of βII spectrin-GFP in low expression and high expression neurons are shown in (**A**) and (**B**), respectively. The STORM image and autocorrelation analyses of boxed regions are shown in (**C**) and (**D**). (**E**) Quantification of the expression levels of βII spectrin-GFP in transfected neurons that show (red) or do not show (black) the periodic lattice structure. The expression levels of the neurons subject to the FRAP analysis in Figure 5D,E are indicated by arrows. **DOI:** http://dx.doi.org/10.7554/eLife.04581.017

The periodic βII spectrin pattern appeared to be mostly static. Fourier analysis of the patterns showed that the spatial frequency (i.e., the period) of the structure did not change over the imaging time of several minutes (Figure 5B). Cross-correlation analysis of the patterns taken at different time points showed no phase shift of the periodic structure during the imaging time (Figure 5C).

As an alternative approach to probe the stability of the structure, we used fluorescence recovery after photo-bleaching (FRAP). For this analysis, we transfected neurons with a βII spectrin-GFP fusion construct, bleached the GFP signal in local regions of axons, and measured the signal recovery rate. In neurons that exhibited moderate expression levels of βII spectrin, where the majority of βII spectrin-GFP molecules were incorporated into the periodic lattice structure (Figure 5—figure supplement 1), the recovery rate was extremely slow and essentially undetectable after 30 min (Figure 5D). In contrast, the fluorescence recovery was much faster (75% recovery in 5 min) in neurons, where the expression level of βII spectrin-GFP was high and the majority of βII spectrin-GFP molecules were not incorporated into the periodic structure (Figure 5E and Figure 5—figure supplement 1). These data indicate that periodic lattice structure was highly stable in live neurons.

### Microtubule dependence of the periodic membrane skeleton

**Figure 6.**
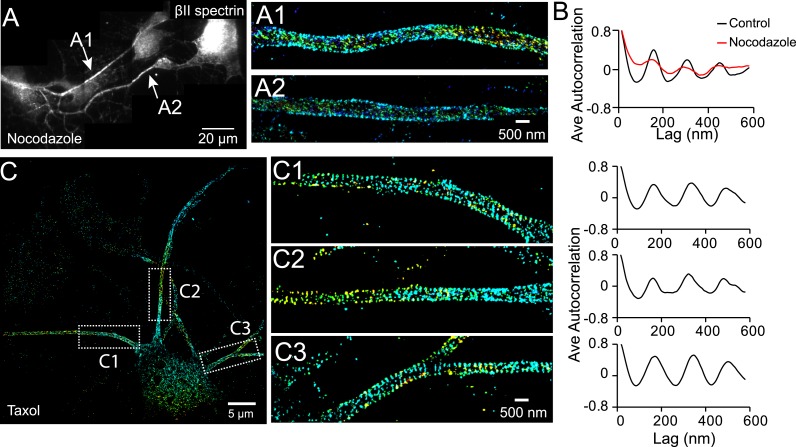
The periodic structure of βII spectrin relies on intact microtubules. (**A** and **B**) DIV 4 neurons were either untreated or treated with nocodazole (50 µM) for 1 hr and immunostained with βII spectrin antibody for 3D STORM. A reconstructed neuron image from nocodazole-treated neurons and two STORM images are shown in **A**. (**B**) Average autocorrelation analyses of βII spectrin from multiple control and nocodazole-treated neurons (n = 9 neurons for control and n = 8 neurons for nocodazole-treated conditions; three independent experiments for each condition). (**C**) Neurons were treated with taxol (5 nM) at DIV 3 for 3 days, which induces the growth of multiple axon-like processes. A representative STORM image of a treated neuron at DIV 6, the enlarged images of the boxed regions, and the autocorrelation analyses (n = 10 neurons, four independent experiments) are shown. **DOI:** http://dx.doi.org/10.7554/eLife.04581.018

Microtubules are essential for the establishment of neuronal polarity. Local stabilization of microtubules is sufficient to induce axon formation (Witte et al., 2008). Moreover, tubulin binds to ankyrin B, a molecule that also interacts with βII spectrin (Bennett and Davis, 1981). We thus tested whether microtubules play a role in the formation of the periodic membrane skeleton structure. In neurons treated with the microtubule-disrupting drug nocodazole (50 µM for 1 hr), the periodic pattern of βII spectrin was largely disrupted (Figure 6A,B). On the other hand, when microtubules were stabilized with taxol (5 nM for 3 days), a treatment that is known to induce multiple axon-like processes in neurons (Witte et al., 2008), we found that βII spectrin exhibited a periodic pattern in all of these axon-like processes (Figure 6C). We also treated neurons with SB 216763, a drug that stabilizes microtubules and promotes axonal growth by inhibiting glycogen synthase kinase-3 beta (GSK-3β) (Jiang et al., 2005; Yoshimura et al., 2005). Similarly, in neurons treated with SB-216763, we observed that the periodic lattice structure was formed in multiple axon-like long processes (Figure 6—figure supplement 1).

**Figure 6—figure supplement 1.**
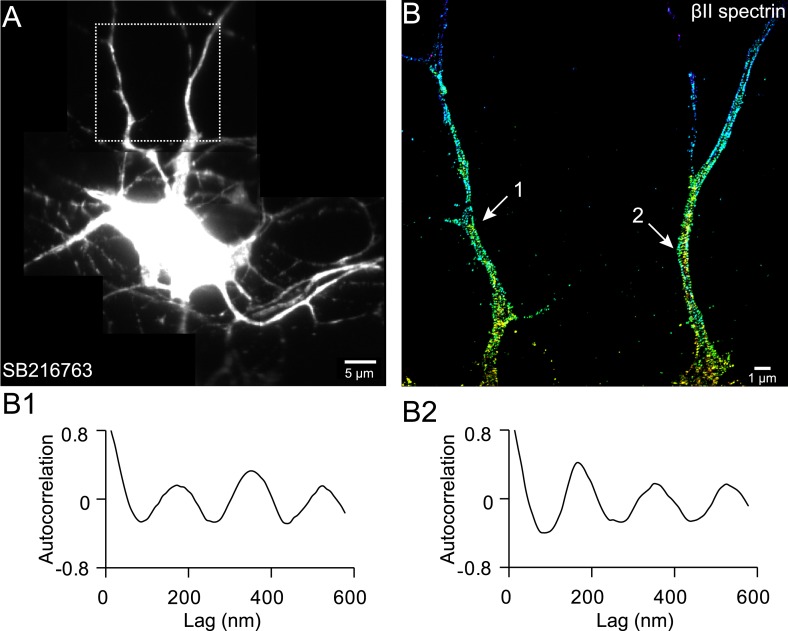
The periodic βII spectrin structure can be induced in multiple processes by axon-promoting small molecules. Neurons were treated with SB 216763 at DIV 1, a drug that inhibits GSK3β and promotes the growth of multiple axon-like processes, and immunostained for βII spectrin at DIV 5. A representative conventional image of βII spectrin (**A**), STORM image of boxed region (**B**), and autocorrelation analyses of the arrow-indicated regions (**B1** and **B2**) are shown. Similar results were found for three independent experiments. **DOI:** http://dx.doi.org/10.7554/eLife.04581.019

### Role of ankyrin B in the periodic membrane skeleton

Given the dramatically different actin–spectrin organizations in axons and dendrites, an interesting question arises as to what molecular factors are critical for promoting the formation of the highly regular, periodic lattice structure in axons and/or suppressing it in dendrites. Ankyrin B (ANK 2) is a molecule that binds to βII spectrin (Bennett and Lorenzo, 2013). It is highly enriched in axons (Chan et al., 1993; Kunimoto, 1995; Engelhardt et al., 2013) and recently found to be potentially linked with autism (De Rubeis et al., 2014; Iossifov et al., 2014). We have shown previously that ankyrin B also adopts a partially periodic pattern in axons albeit with a lower regularity (Xu et al., 2013), potentially due to an incomplete occupancy of the ankyrin B binding sites on the lattice structure and the presence of ankyrin B on intracellular membranes (Bennett and Lorenzo, 2013). We thus asked whether ankyrin B is involved in regulating the formation of this periodic membrane skeleton in axons.

**Figure 7.**
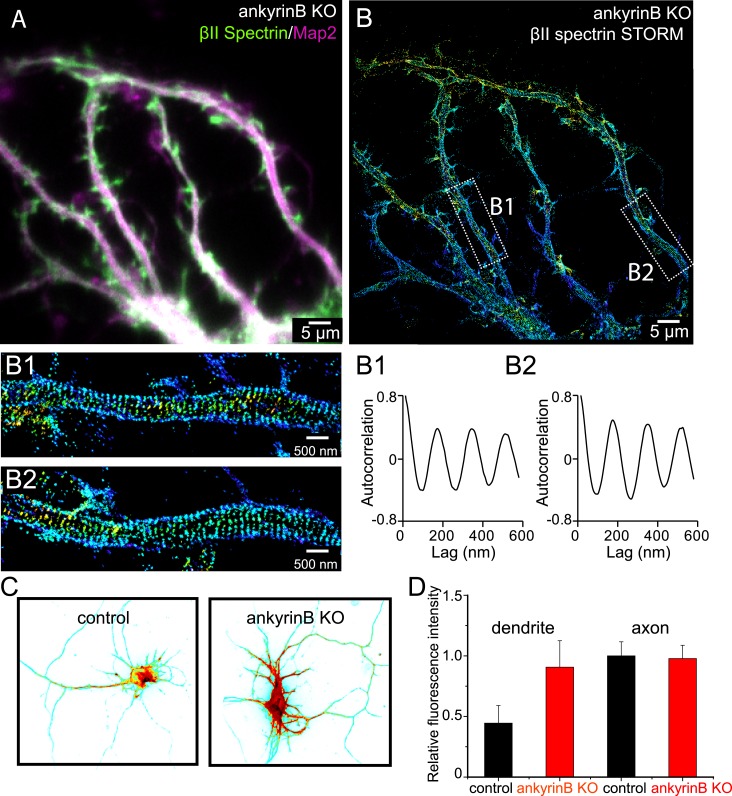
Role of ankyrin B in the regulation of the periodic lattice structure. (**A**–**B**) DIV 10 neurons from ankyrin B knockout (KO) mice were immunostained for βII spectrin and a dendritic marker Map2 and imaged. (**A**) Conventional image of βII spectrin and Map2. (**B**) 3D STORM of βII spectrin. The image is taken from the green-boxed region of the neuron in Figure 7—figure supplement 1. The enlarged STORM image and autocorrelation analyses of boxed regions are shown in (**B1**) and (**B2**). Similar results were found in four independent experiments. (**C**) Conventional βII spectrin image from wild-type (control) and ankyrin B KO DIV 10 neurons. The fluorescence intensity is coded by color, with red indicating higher expression. (**D**) The relative fluorescence intensity of βII spectrin in dendrites and axons of wild-type and ankyrin B KO neurons (n = 14 neurons for wild-type and n = 13 neurons for ankyrin B KO; three independent experiments). **DOI:** http://dx.doi.org/10.7554/eLife.04581.020

To address this question, we performed STORM imaging on disassociated hippocampal neurons from ankyrin B knockout mice (Scotland et al., 1998) at DIV 10. Similar to wild-type neurons, ankyrin B knockout neurons still showed enrichment of MAP2 in dendrites with similar dendritic morphology, making dendrites easy to identify in these neurons (Figure 7A and Figure 7—figure supplement 1). The periodic pattern of βII spectrin in axons was not perturbed by ankyrin B deletion and appeared quantitatively similar to that observed in control wild-type neurons (Figure 7—figure supplement 1). Surprisingly, βII spectrin also adopted a highly regular, periodic distribution in all dendrites, with the periodicity quantitatively similar to that observed in axons (Figure 7A,B). This is in stark contrast to what we observed in wild-type neurons, where the distributions of βII spectrin in dendrites were largely irregular (Figure 1—figure supplement 5). The actin-capping protein adducin also adopted a periodic distribution in dendrites of ankyrin B knockout neurons, with quantitatively similar periodicity to that of βII spectrin. Knocking-down βII spectrin disrupted the periodic distribution of adducin, indicating that the periodic lattice structure in the dendrites of the ankyrin B knockout neurons also depended on βII spectrin (Figure 7—figure supplement 2). These data indicate that the formation of the periodic membrane skeleton does not require ankyrin B. Instead, ankyrin B is important for inhibiting the formation of this periodic lattice structure in dendrites.

**Figure 7—figure supplement 1.**
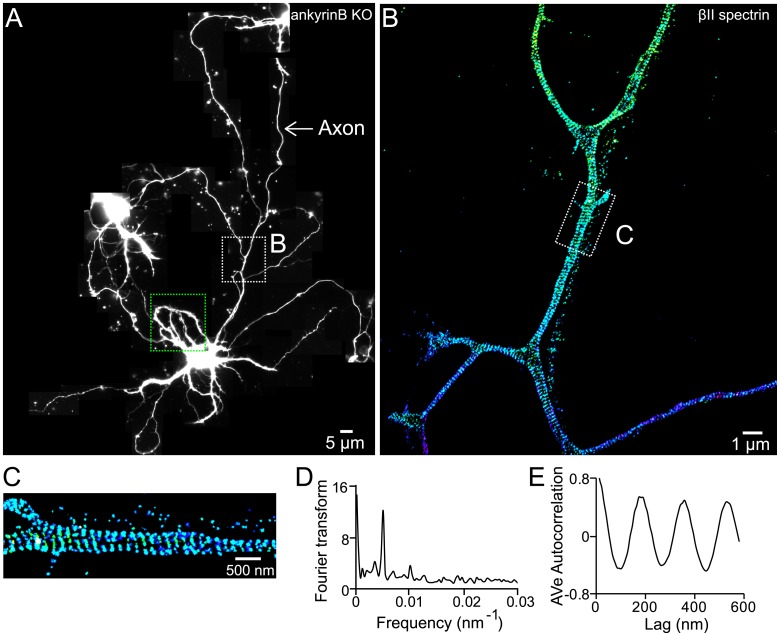
The βII spectrin structure in axons of ankyrin B KO neurons. (**A**) Reconstructed image of an ankyrin B KO DIV 10 neuron. βII spectrin was subjected for 3D STORM image in both dendrites and axons. The STORM image of dendrites in green-boxed region is shown in Figure 7. The STORM image of axon in white-boxed region is shown in (**B**). (**C**) Enlarged STORM image of the boxed region in (**B**). (**D**–**E**) Fourier transform and autocorrelation analyses of the axon segment shown in **C**. **DOI:** http://dx.doi.org/10.7554/eLife.04581.021

**Figure 7—figure supplement 2.**
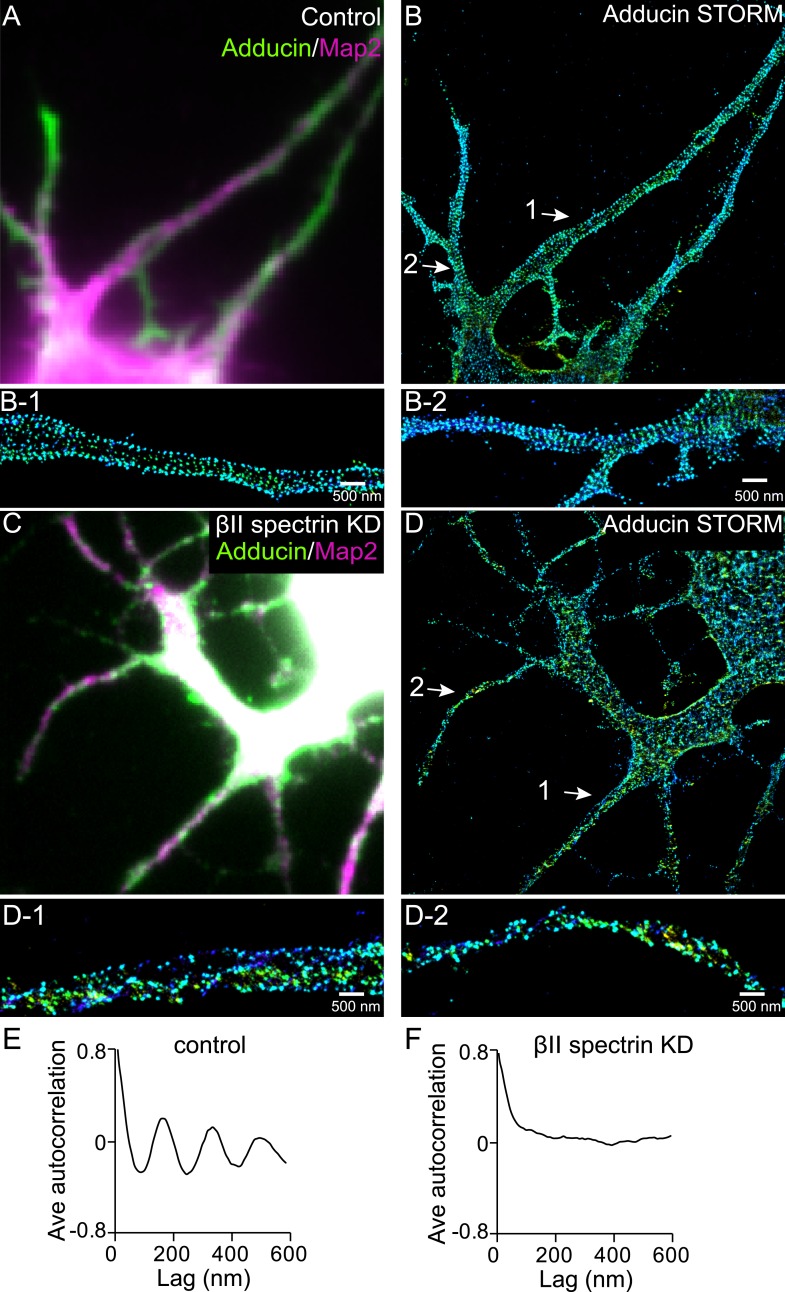
Formation of the periodic lattice structure in dendrites of ankyrin B knockout neurons depends on βII spectrin. (**A**–**B**) Ankyrin B knockout neurons were immunostained for adducin (green) and MAP2 (magenta), and imaged by conventional (**A**) and 3D STORM (**B**) microscopy. Magnified STORM images of adducin for arrow-indicated regions are shown in (**B-1**) and (**B-2**). (**C**–**D**) Ankyrin B knockout neurons were infected with βII spectrin-shRNA expressing adenovirus, immunostained for adducin and MAP2, and imaged by conventional (**C**) and 3D STORM (**D**) microscopy. Infected neurons were marked by GFP signal expressed from the virus (not shown). Magnified STORM images of adducin from arrow-indicated region are shown in (**D-1**) and (**D-2**). (**E**) Average autocorrelation analysis of adducin distribution in dendrites of ankyrin B knockout neurons (n = 9 neurons, four independent experiments). (**F**) Average autocorrelation analysis of adducin distribution in dendrites of ankyrin B knockout neurons treated with βII spectrin-shRNA expressing adenovirus (n = 15 neurons, three independent experiments). **DOI:** http://dx.doi.org/10.7554/eLife.04581.022

### Local βII spectrin concentration regulates the formation of this periodic lattice structure, and ankyrin B regulates the polarized distribution of βII spectrin in neurites

In addition to the induction of the periodic lattice structure in dendrites, we noticed that ankyrin B knockout also induced a dramatic redistribution of βII spectrin in neurites. In wild-type neurons, the local concentration of βII spectrin, as indicated by immunofluorescence intensity, was ∼twofold higher in axons than that in dendrites (Figure 7C,D), consistent with previous results (Riederer et al., 1986; Galiano et al., 2012). However, the expression level of βII spectrin was substantially increased in dendrites by the ankyrin B knockout to a point that the local concentration of βII spectrin in dendrites became indistinguishable from that in axons, and both were comparable to the βII spectrin concentration observed in wild-type axons (Figure 7C,D). We thus hypothesized that the increased local concentration of βII spectrin caused the formation of this periodic lattice structure in dendrites.

**Figure 8.**
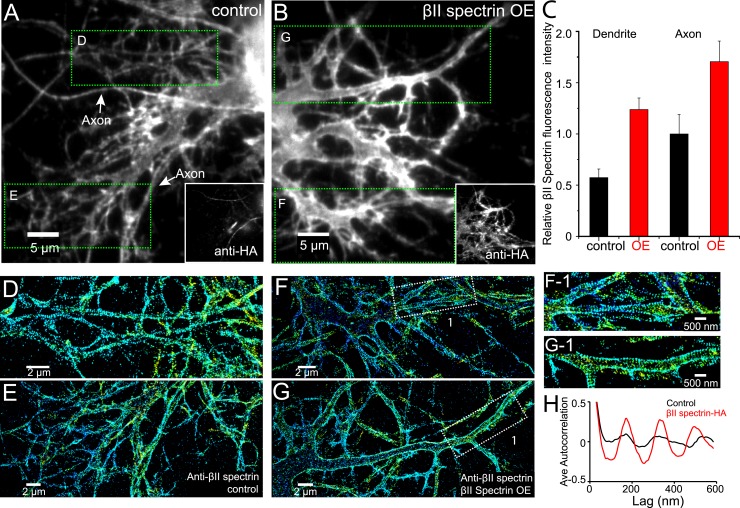
Local βII spectrin concentration determines the formation of the periodic lattice structure. DIV 9 neurons were either mock-transfected or transfected with βII spectrin-HA and immunostained for HA and βII spectrin. βII spectrin were subsequently imaged by 3D STORM. (**A**–**B**) Conventional images of βII spectrin in dendrites of a control neuron and a βII spectrin-HA overexpressing (OE) neuron. The HA image is shown in the insets. (**C**) The relative fluorescence intensity for βII spectrin in dendrites and axons of control and βII spectrin-HA overexpressing neurons (n = 10 neurons for control and n = 15 neurons for βII spectrin-HA overexpressing conditions; three independent experiments for each condition). (**D**–**E**) STORM images of βII spectrin from green-boxed regions in **A**. (**F**–**G**) STORM images of βII spectrin from green-boxed regions in **B**. (**F-1**) and (**G-1**) are enlarged images of the white-boxed regions in **F** and **G**, respectively. (**H**) Average autocorrelation analyses of βII spectrin in dendrites of control and βII spectrin-HA overexpressing neurons (n = 8 neurons for control and n = 12 neurons for βII spectrin-HA overexpressing conditions; three independent experiments for each condition). **DOI:** http://dx.doi.org/10.7554/eLife.04581.023

To test this hypothesis, we increased the expression level of βII spectrin in all neurites by transiently transfecting neurons with a HA-tagged βII spectrin construct and performed STORM imaging on βII spectrin in transfected neurons at DIV 11. As expected, the local concentration of βII spectrin in the dendrites of βII spectrin-HA expressing neurons was higher than that observed in control neurons that did not express βII spectrin-HA (Figure 8A–C). Remarkably, whereas βII spectrin appeared mostly irregular in the dendrites of control neurons (Figure 8D,E), in βII spectrin-HA overexpressing neurons, βII spectrin displayed a periodic pattern in nearly all dendritic processes (Figure 8F,G). Autocorrelation analysis showed that the periodicity in the dendrites of overexpressing neurons was substantially enhanced compared to the dendrites of control neurons (Figure 8H).

Taken together, these data suggest that the local concentration of βII spectrin is a key determining factor for the formation of the periodic membrane skeleton in axons and that ankyrin B was critical for setting the polarized distribution of βII spectrin in axons and dendrites.

## Discussion

Actin, spectrin, and associated molecules form a periodic lattice structure with long-range order underneath the axonal membrane. Many molecular components, including actin, βII spectrin, adducin, ankyrin B, βIV spectrin, ankyrin G, and sodium channels, are present in this structure. We have observed this periodic membrane skeleton with STORM imaging of fixed cultured neurons (Figure 1 and Figure 1—figure supplements 1–3) and fixed brain tissue slices (Xu et al., 2013) using actin-binding phalloidin and immunolabeling of endogenously expressed proteins, as well as in live cultured neurons using fluorescent fusion proteins (Figure 5). Recently, this periodic structure has also been observed in live neurons using a cell-permeable actin-binding dye and STED imaging (Lukinavicius et al., 2014). Since a single actin filament can interact with multiple spectrin tetramers, and a single spectrin tetramer can bind to two actin filaments, one at each end of the symmetric tetramer (Bennett and Lorenzo, 2013), we reason that these crosslinking interactions are responsible for the formation of the lattice structure. Indeed, depolymerizing actin filaments disrupted the periodic distribution of βII spectrin, and knocking-down of βII spectrin disrupted the periodic distribution of actin (Figure 1—figure supplements 2–3). Because this lattice structure is associated with the axonal membrane, proper preservation of the membrane structure is essential for observing this structure. For example, a recent electron microscopy study of the AIS in neurons that has been subjected to detergent extraction of membrane before fixation did not show such periodic membrane skeleton (Jones et al., 2014). Indeed when we applied the same ‘fixation after membrane extraction’ protocol to neurons, the structure was destroyed and not observed in STORM images (Figure 1—figure supplement 6). In this study, we investigated the developmental mechanism of this newly discovered axonal membrane skeleton.

We found that this periodic membrane skeleton started to form early during axon development. In stage 3 neurons at DIV 2, when one neurite just broke the symmetry and became an axon (typically several times longer than other neurites), the periodic pattern of βII spectrin already emerged (Figure 1). It originated in the proximal axon regions near the cell bodies and gradually propagated to the distal ends of axons (Figure 1). This spatial distribution is in contrast to most previously identified signaling molecules involved in axon differentiation and development, which are enriched and function at the growing tip of axons (Arimura and Kaibuchi, 2007; Barnes and Polleux, 2009; Stiess and Bradke, 2011; Cheng and Poo, 2012). After its initial appearance, the lattice structure continued to mature with the actin filaments becoming more stable, potentially because of capping by adducin (Figures 2, 3). Once matured, the structure appeared highly stable with little movement and extremely slow turnover of its molecular components was observed in live neurons (Figure 5). This highly stable membrane skeleton may function to provide a stable mechanical support for axons. Indeed, deletion of β spectrin from *C elegans* causes axons to break when the animals move (Hammarlund et al., 2007).

The emergence of this periodic lattice during early axon development and its origination in the proximal axon region near the cell body suggest that the periodic membrane skeleton may function as an independent mechanism for establishing or maintaining neuronal polarization in addition to the previously identified pathways that function at the distal ends of axons. However, the periodic membrane skeleton is not required for the initiation of axon differentiation because the periodic structure only started to form in stage 3, but not stage 2, a stage at which most signaling molecules for axon initiation exhibit high activity (Arimura and Kaibuchi, 2007; Cheng and Poo, 2012). Moreover, neurons depleted of βII spectrin are also capable of forming long axons (Galiano et al., 2012), though these axons may not be fully functional. Indeed, removal of αII or βII spectrin is embryonically lethal in mice (Tang et al., 2003; Stankewich et al., 2011) and AIS, the structure important for action potential generation, fails to assemble properly in neurons that are depleted of βII spectrin (Galiano et al., 2012).

Interestingly, molecules important for the specification of AIS, ankyrin G, βIV spectrin, and sodium channels were all incorporated into this periodic membrane skeleton. During the early stages of axon development, the expression levels of ankyrin G and βIV spectrin were low in the proximal axon region, which was instead occupied by the periodic lattice comprising βII spectrin (Figure 1). Ankyrin G and βIV spectrin began to enrich in the proximal axon region later during axon development, at ∼DIV 8 (Figure 4—figure supplement 1), consistent with previous observations (Galiano et al., 2012). The incorporation of ankyrin G and βIV spectrin into the periodic lattice was observed even later, at around DIV 12, replacing βII spectrin from the structure in the AIS (Figure 4). The enrichment and periodic assembly of the AIS components appeared to depend on βII spectrin (Figure 4—figure supplement 2). Our data suggest the following developmental course for the AIS formation. Before the AIS is formed, actin and βII spectrin form a cohesive periodic lattice structure that covers the entire axonal shaft including the proximal axonal region. The AIS then forms by the enrichment of ankyrin G and βIV spectrin in the proximal axonal region and the replacement of βII spectrin by βIV spectrin in the periodic membrane skeleton. Potentially, ankyrin G, the master regulator of AIS that recruits other AIS components (Zhou et al., 1998; Jenkins and Bennett, 2001; Yang et al., 2007), is first enriched in the proximal axon region. Ankyrin G then recruits βIV spectrin to the same region and causes it to be incorporated in the periodic lattice. βIV spectrin in turn anchors ankyrin G into a periodic pattern as well. As an adaptor, ankyrin G then places sodium channels into a periodic distribution pattern in the AIS.

Finally, we addressed the question why the cohesive, periodic lattice structure preferentially formed in axons, with dendrites showed at most isolated patches of periodic structure with much less regularity (Figure 1—figure supplement 5). We found multiple molecular factors participate in this regulation. The periodic lattice structure depended on intact microtubules. Treatment with a microtubule-depolymerizing drug disrupts the structure in axons, whereas treatment with microtubule-stabilizing drugs induces the formation of the lattice structure in multiple neurites (Figure 6). Importantly, we found that the local concentration of βII spectrin is a determining factor for the formation of the lattice structure. The local concentration of βII spectrin is ∼2 times higher in axons than in dendrites (Figure 7). Remarkably, increasing the dendritic concentration of βII spectrin by overexpression induced the formation of the periodic lattice structure in all dendrites (Figure 8). Interestingly, ankyrin B was critical for maintaining the polarized distribution of βII spectrin. Knocking out ankyrin B led to an even distribution of βII spectrin in dendrites and axons and the formation of a highly regular and cohesive periodic lattice structure in all dendrites (Figure 7). Consistent with the notion that the increased concentration of βII spectrin in dendrites is responsible for inducing the formation of the periodic lattice structure in dendrites, knocking-down βII spectrin from ankyrin B knockout neurons disrupted the lattice structure (Figure 7—figure supplement 2). These results indicate that ankyrin B is critical for establishing a polarized distribution of βII spectrin in neurites with a higher concentration of βII spectrin in axons than in dendrites, which in turn promotes the formation of the periodic membrane skeleton in axons.

It is interesting to speculate how ankyrin B may establish such a polarized distribution of βII spectrin. The predominant form of ankyrin B during early neuronal development is a 440-kDa splice variant that is preferentially targeted to axons (Kunimoto et al., 1991; Chan et al., 1993; Kunimoto, 1995). Given that ankyrin B specifically binds to βII spectrin, we speculate that the distribution of ankyrin B in axons may help establishing the enrichment of βII spectrin in axons during early neuronal development. Moreover, we recently found that ankyrin B is a major cargo adaptor for dynactin and promotes axonal transport of proteins and organelles and that disruption of ankyrin B–dynactin interaction significantly impairs the axonal transport of many proteins (Lorenzo et al., 2014). It is thus possible that ankyrin B may also preferentially transport βII spectrin into axons instead of dendrites. By maintaining a polarized distribution of βII spectrin in neurons, ankyrin B functions as a negative regulator for preventing the formation of the periodic membrane skeleton in dendrites. The exact mechanism by which ankyrin B maintains a polarized distribution of βII spectrin remains an interesting question for future investigation.

## Materials and methods

All experimental procedures were performed in accordance with the Guide for the Care and Use of Laboratory Animals of the National Institutes of Health. The protocol was approved by the Institutional Animal Care and Use Committee (IACUC) of Harvard University.

### Neuron culture

Primary hippocampal cultures were prepared from wild-type neonatal (E18) rat embryos (timed pregnant SD rats from Charles River Laboratories, Wilmington, MA) or ankyrin B knockout mice as reported previously (Scotland et al., 1998). Hippocampi were isolated and digested with 0.05% trypsin–EDTA (1×) (Invitrogen 25300-054, Grand Island, NY) at 37°C for 15 min. The hippocampi were transferred to the Hib A solution (BrainBits HA-Ca, Springfield, IL), washed several times with the Hib A solution, and pipetted up and down until the tissues were mostly dissolved. The solution was then passed through a cell strainer (VWR 21008-949, Philadelphia, PA) to remove the residual undissociated tissue and collected in a 50 ml conical tube. Neurons were spun down to the bottom of the tube, resuspended with the culture media made of 96 ml Neurobasal (Life Technologies 12349-015), 2 ml B-27 Supplement (Life Technologies 17504-044, Grand Island, NY), 1 ml Penicillin-Streptomycin (Life technologies 15140-122) and 1 ml Glutamax (Life technologies 35050-061), and then plated onto poly-L-lysine/laminin-coated 12-mm coverslips (BD bioscience BD354087, San Jose, CA) or poly-L-lysine coated 8-well chambers. 5 μM cytosine-D-arabinofuranoside (Sigma C1768, St. Louis, MO) was added to the culture media to inhibit the growth of glial cells 3 days after plating. The neurons were fed twice a week with freshly made culture media until use.

### Reagents

The following primary antibodies were used in this study: guinea pig anti-Map2 antibody (Synaptic Systems, 188002, Goettingen, Germany); mouse anti-βII spectrin antibody (BD Biosciences, 612563); mouse anti-ankyrin G antibody (Santa Cruz, Sc-12719, Dallas, Texas; epitope mapping the spectrin-binding domain of ankyrin G near the N-terminal); goat anti-ankyrin G antibody (Santa Cruz, Sc-31778, epitope mapping the C-terminal of ankyrin G); rabbit anti-adducin antibody (Abcam, ab51130, Cambridge, MA); rabbit anti-HA antibody (Abcam, ab9110); rabbit anti-GFP antibody (Abcam, ab290); goat anti-βIII spectrin (Santa Cruz, sc-9660). Rabbit antibodies targeting the C- or N-terminus of βIV spectrin were kind gifts from Dr Matt Rasband at Baylor College of Medicine.

The following secondary antibodies were used in this study for conventional imaging: Alexa Fluor 647 donkey anti-mouse (Invitrogen, A31571), Alexa Fluor 555 donkey anti-mouse (Invitrogen, A31570), Alexa Fluor 488 donkey anti-mouse (Invitrogen, A21202), Alexa Fluor 647 donkey anti-rabbit (Invitrogen, A31573), Alexa Fluor 568 donkey anti-rabbit (Invitrogen, A10042), Alexa Fluor 488 donkey anti-rabbit (Invitrogen, A21206), Alexa Fluor 488 goat anti-guinea pig (Invitrogen, A11073), Alexa Fluor 647 donkey anti-goat (Invitrogen, A21447), Alexa Fluor 568 donkey anti-goat (Invitrogen, A11057).

For STORM imaging, secondary antibodies were custom-labeled with a photoswitchable reporter dye, Alexa Fluor 647, and an activator dye Alexa Flour 405, which facilitates the photoswitching of the reporter dye. Donkey anti-mouse and donkey anti-rabbit secondary antibodies (Jackson ImmunoResearch, West Grove, PA) were each labeled with a mixture of amine-reactive activator and reporter dyes in a one-step reaction, as described previously (He et al., 2013). In some experiments, commercial Alexa Fluor 647 conjugated donkey anti-mouse, anti-rabbit or anti-goat secondary antibodies were used.

### Transfection of neurons with fusion protein constructs

βII spectrin-GFP and βII spectrin-HA plasmids (addgene, 31070, Cambridge, MA) used in this study were reported previously, with GFP inserted at the N-terminal of βII spectrin and the HA tag at the C-terminal of βII spectrin, respectively (Galiano et al., 2012). To make the βII spectrin-mMaple 3 construct, we replaced the HA tag sequence with the mMaple 3 sequence (Wang et al., 2014). Plasmids were transfected into neurons using a calcium phosphate transfection kit from Invitrogen (K2780-01). The protocol for transfection was modified slightly for our neuronal cultures. Briefly, neurons were plated at a density of 40,000 cells/well in 12-well plates and cultured for 6–10 days before the transfection. After changing media from original neuronal culture media to Minimum Essential Media (MEM, Life Technology, supplemented with 20 mM HEPEs, pH 7.15), 100 µl plasmid mixture was added, and neurons were incubated at 37°C for 20 min. The media was subsequently aspirated and replaced with a lower pH MEM (supplemented with 20 mM HEPEs, pH 6.8) at 37°C for 4 min. After dissolving all calcium phosphate crystals, we added back the original neuronal culture media. Experiments were performed 2 or 3 days after transfection.

### Knockdown with shRNA

The βII spectrin-shRNA adenoviral construct used in this study was a kind gift from Dr Matt Rasband at Baylor College of Medicine and described previously (Hedstrom et al., 2008). The two sense sequences of shRNA are: 5′-GCATGTCACGATGTTACAA-3′ and 5′-GGATGAAATGAAGGTGCTA-3′. For assaying the effect of βII spectrin knockdown on actin and adducin structure, neurons were infected with the virus at DIV 3 and fixed for STORM imaging at around DIV 9 or 10. For assaying the effect of βII spectrin knockdown on βIV spectrin structure, neurons were infected with the virus at DIV 3 or DIV 7 and fixed at DIV 12 for STORM imaging of βIV spectrin. Infected neurons were marked by a GFP signal expressed from the adenoviral construct. The knockdown efficiency was validated through immunostaining against βII spectrin.

### Drug treatment of neurons

The following chemicals were used in this study with their concentration and treatment time stated: latrunculin A (Sigma, L5163, 20 µM, 1 hr or indicated time series), cytochalasin D (Sigma, C8273, 50 µM, 1 hr), nocodazole (Sigma, M1404, 50 µM, 1 hr), taxol (Sigma, T7402, 5 nM), SB-216763 (Sigma, S3442, 5 µM). For taxol treatment, neurons were treated with the drug at DIV 3 with the indicated concentration and fixed at DIV 6 for STORM imaging. For SB216763 treatment, neurons were treated with the drug at DIV 1 with the indicated concentration and fixed at DIV 5 for STORM imaging.

### Fluorescence labeling of neurons

Cultured neurons were fixed at various days in vitro (DIV). For imaging of actin, we used a similar method to label actin as reported previously (Koestler et al., 2008; Xu et al., 2013). Briefly, the samples were simultaneously fixed and extracted for 1 min using a solution of 0.3% (vol/vol) glutaraldehyde (GA) and 0.25% (vol/vol) Triton X-100 in cytoskeleton buffer (CB, 10 mM MES, pH 6.1, 150 mM NaCl, 5 mM EGTA, 5 mM glucose, and 5 mM MgCl_2_) and then post-fixed for 15 min in 2% (vol/vol) GA in CB, a previously established protocol for maintaining actin ultrastructure (Koestler et al., 2008; Xu et al., 2013). The GA-fixed samples were treated with freshly prepared 0.1% (wt/vol) sodium borohydride for 7 min to reduce background fluorescence caused by GA fixation. To label actin filaments, samples were labeled with Alexa Fluor 647 conjugated phalloidin (Invitrogen A22287) overnight at 4°C or ∼1 hr at room temperature. A concentration of ∼0.5 µM phalloidin in PBS was used. To minimize the dissociation of phalloidin from actin during washing steps, actin labeling was performed after all other labeling steps (i.e., immunofluorescence of other molecular targets) were completed. The sample was washed 2–3 times with PBS and then immediately mounted for imaging.

To test whether strong membrane extraction prior to fixation (Jones et al., 2014) disrupts the membrane skeleton structure, neurons were extracted with 1% Triton X-100 in PEM buffer (100 mM Pipes–KOH, pH 6.9, 1 mM MgCl_2_, and 1 mM EGTA) containing 2% polyethylene glycol, 2 µM phalloidin, and 2 µM taxol for 3 min at room temperature after a quick rinse with the PEM buffer containing 2 µM taxol and subsequently fixed with 0.2% GA in PBS for at least 20 min, as described previously (Jones et al., 2014). Fixed samples were treated with freshly prepared 0.2% (wt/vol) sodium borohydride for 5–10 min to reduce background fluorescence caused by GA fixation and washed in PBS. Actin labeling was performed similarly as described above.

For imaging of molecular components not including actin (MAP2, βII spectrin, βIII spectrin, βIV spectrin, ankyrin G, and adducin), the samples were fixed using 4% (wt/vol) paraformaldehyde in phosphate buffered saline (PBS) for 15 min. Fixed neuron samples were then permeabilized and blocked in blocking buffer (3% wt/vol bovine serum albumin or 10% wt/vol donkey serum, 0.2% vol/vol Triton X-100 in PBS) for 1 hr and subsequently stained with primary antibodies in blocking buffer overnight at 4°C. The samples were washed three times and then stained with secondary antibodies (described above) in blocking buffer for ∼1 hr at room temperature.

### Fixed-cell STORM imaging

The imaging buffer was PBS containing 100 mM cysteamine, 5% glucose, 0.8 mg/ml glucose oxidase (Sigma-Aldrich), and 40 μg/ml catalase (Roche Applied Science, Indianapolis, IN) for fixed neurons. To image the samples from 12-mm coverslips, approximately 4 μl of imaging buffer was dropped at the center of a freshly-cleaned #1.5 rectangular coverslip (22 mm by 60 mm), and the sample on the 12-mm coverslip was mounted on the rectangular coverslip and sealed with nail polish or Cytoseal. To image samples from 8-well chambers, 400 μl of imaging buffer was added to the imaging chamber.

The STORM setup was based on an Olympus IX-71 inverted optical microscope as described previously (Jones et al., 2011). 405-nm (CUBE 405-50C; Coherent, Santa Clara, CA), 460-nm (Sapphire 460-10; Coherent), 532-nm (GCL-200-I; CrystaLaser, Reno, NV), and 657-nm (RCL-300-656; CrystaLaser) lasers were introduced into the sample through the back focal plane of the microscope. A translation stage allowed the laser beams to be shifted towards the edge of the objective so that the emerging light reached the sample at incidence angles slightly smaller than the critical angle of the glass–water interface, thus illuminating only the fluorophores within a few micrometers of the coverslip surface. A T660LPXR (Chroma, Bellows Falls, VT) was used as the dichroic mirror and an ET705/72M band-pass filter (Chroma) was used as the emission filter. For 3-dimensional (3D) STORM imaging, a cylindrical lens was inserted into the imaging path so that images of single molecules were elongated in *x* and *y* for molecules on the proximal and distal sides of the focal plane (relative to the objective), respectively (Huang et al., 2008).

During imaging, continuous illumination of 657-nm laser (∼2 kW/cm^2^) was used to excite fluorescence from Alexa Flour 647 molecules and switched them into the dark state. Continuous illumination of the 405-nm laser (when Alexa Flour 405 was used as the activator dye) or 532-nm laser (when Cy3 was used as the activator dye) was used to reactivate the fluorophores to the emitting state. The power of the activation lasers (typical range 0–1 W/cm^2^) was adjusted during image acquisition so that at any given instant, only a small, optically resolvable fraction of the fluorophores in the sample was in the emitting state.

A typical STORM image was generated from a sequence of about 30,000–60,000 image frames at a frame rate of 60 Hz. The recorded STORM movie was analyzed according to previously described methods (Rust et al., 2006; Huang et al., 2008). The centroid positions and ellipticities of the single molecule images provided lateral and axial positions of each activated fluorescent molecule, respectively (Huang et al., 2008). Super-resolution images were reconstructed from the molecular coordinates by depicting each location as a 2D Gaussian peak.

### Live-cell STORM imaging

Live-cell STORM experiments were performed on the same STORM setup as described earlier (Jones et al., 2011; Shim et al., 2012). Neurons were initially transfected with βII spectrin-mMaple 3 at DIV 8 and imaged in an extracellular solution containing: 128 mM NaCl, 5 mM KCl, 2 mM CaCl_2_, 1 mM MgCl_2_, 25 mM HEPES, 30 mM glucose, pH 7.3 at DIV 10 or DIV 11. Continuous illumination of the 405-nm laser was used to activate the mMaple 3 fluorescent protein. Continuous illumination of 561-nm laser was used to excite mMaple 3 and switched them to the dark state. Imaging analysis was performed as described above.

### FRAP analysis

FRAP experiments were performed on the same STORM setup as described above. Neurons were transfected with βII spectrin-GFP at DIV 8 and then imaged at DIV 10. Directly before FRAP experiments, neuronal culture media were replaced with an extracellular solution. After recording an image before photo-bleaching, a small region of the sample was bleached by shrinking the size of iris at the excitation light path for 10 s with the maximum laser power. Subsequently, the sample was imaged using the same power as that of pre-bleached image. The image was recorded at a frequency of 1 Hz. We used the unbleached regions in the image to calibrate the photo-bleaching effect during the entire recording time. The fluorescence recovery fraction was measured as the fluorescence intensity at the bleached region at indicated time vs the original intensity before photo-bleaching.
