# Supplementary material for: Developmental mechanism of the periodic membrane skeleton in axons
Source: eLife. 2014 Dec 23;3:e04581. doi: 10.7554/eLife.04581 (PMC4337613; doi:10.7554/eLife.04581)
Supplement: Related texts

# Article commentary: Decision letter

**DOI:** 10.7554/eLife.04581.024

**Affiliations:** Albert Einstein College of Medicine, United States

eLife posts the editorial decision letter and author response on a selection of the published articles (subject to the approval of the authors). An edited version of the letter sent to the authors after peer review is shown, indicating the substantive concerns or comments; minor concerns are not usually shown. Reviewers have the opportunity to discuss the decision before the letter is sent (see review process). Similarly, the author response typically shows only responses to the major concerns raised by the reviewers.

Thank you for sending your work entitled “Developmental mechanism of the periodic membrane skeleton in axons” for consideration at *eLife*. Your article has been favorably evaluated by Eve Marder (Senior editor) and 3 reviewers, one of whom is a member of our Board of Reviewing Editors. All reviewers were very laudatory about your manuscript, and feel it will be a valuable and important contribution.

The Reviewing editor and the other reviewers discussed their comments before we reached this decision, and the Reviewing editor has assembled the following comments to help you prepare a revised submission.

The manuscript “Developmental mechanism of the periodic membrane skeleton in axons” by Zhong et al. provides new insights how the lately discovered periodic lattice structure in axons containing actin and spectrin is formed. The authors showed that the membrane skeleton forms early on during neuronal differentiation, when axons are formed. Components of the axon initial segment, ankyrin G and βIV spectrin were integrated into the membrane skeleton late during development. βII spectrin was a positive regulator of membrane skeleton formation and ankyrin B was a negative regulator of membrane skeleton formation. In fact, overexpression of βII spectrin or knocking out of ankyrin B induced the formation of a periodic membrane skeleton in dendrites.

The Zhuang lab has recently made a big contribution to the cytoskeletal field by discovering that axons contain a periodic actin-spectrin based network (Xu et al., Science, 2013). Here, the authors reveal how the periodic skeleton is formed. It is a very exciting work that is suitable for a general audience. In addition, the authors demonstrate that detergent treatment causes the disruption of the periodic membrane skeleton, which shows why recent work from the Svitkina lab were unable to find a periodic membrane skeleton by EM. Before publication, the following points should be addressed.

1) The authors should clearly state, for each experiment, how many neurons they analyzed from how many independent preparations.

2) Please discuss potential mechanisms how ankyrin B, which is localized to the axon, can prevent formation of the lattice in dendrites.

3) Does the morphology of the dendrite change when the lattice is formed there, either by knocking out ankyrin B or by overexpression of βII spectrin? From the pictures, it looks like they are still thicker than axons and a tapered structure. Here a quantification would be important.

4) Figure 1: the presented neuron is rather atypical for a neuron 10 days in culture. They have very short dendrites, more reminiscent of immature minor neuritis (please check Gomis-Rüth et al., Nature Protocols, 2014). Please provide an example of more mature dendrites, and show the absence of the skeleton compared to the axon.

5) In Figure 1A, for images in A1, A2 and A3, how are the exposure times controlled for these images? Is it possible that the periodical distribution is already present in A2 but with less spectrin at each ring. As a result, when given the same exposure time as A1, less photo switching took place. In other words, is it possible that A2 is a partial pattern of A1?

6) The earliest developmental stages that actin and spectrin rings can be detected are different. This is a bit troublesome to reconcile with the complete interdependence between actin and spectrin. The explanation given by the authors is that the actin rings might be lost during the fixation and staining procedures. Why not do these experiments with live neurites and mMaple labeled actin, which would bypass the fixation problem?

7) It is not clear how ankyrin B prevents the localization of βII spectrin in dendrites. To prove that βII spectrin is responsible for the actin rings in the dendrites of ankyrin B knockout cells, the authors could knockdown βII spectrin in the ankyrin B mutant cells.

8) It is not clear from the writing how microtubules contribute to the actin rings. The rationale of the microtubule experiments should be spelled out more clearly.

9) The invasion of the actin rings in the dendrites in the ankyrin B mutant cells provides the authors with the opportunity to understand the functional importance of these rings. Are there any morphological changes of the dendrites or defects in axon-dendrite polarity in these knockout neurons?

# Reply: Author response

**DOI:** 10.7554/eLife.04581.025

*1) The authors should clearly state, for each experiment, how many neurons they analyzed from how many independent preparations*.

*As suggested, we have included the numbers of analyzed neurons and independent experiments in the revised manuscript (please see revised figure captions)*.

*2) Please discuss potential mechanisms how ankyrin B, which is localized to the axon, can prevent formation of the lattice in dendrites*.

Our experimental results showed that the local concentration of βII spectrin, which is higher in axons than in dendrites, is critical for the preferential formation of the periodic lattice structure in axons. We envision two potential mechanisms by which ankyrin B could lead to a higher local concentration of βII spectrin in axons and thereby function as a negative regulator for preventing the formation of the periodic structure in dendrites. We discussed these mechanisms in the last paragraph of the Discussion section.

*3) Does the morphology of the dendrite change when the lattice is formed there, either by knocking out ankyrin B or by overexpression of βII spectrin? From the pictures, it looks like they are still thicker than axons and a tapered structure. Here a quantification would be important*.

In light of this comment, we quantified the morphology of dendrites by two measures, the average length of the dendrites and the diameter variation along individual dendrites. We measured these quantities for both wild type and ankyrin B knockout neurons at DIV9. For comparison, we measured the same quantities for axons. The results are shown below in Author response image 1A-C.

We didn’t observe a significant difference in the average dendritic length upon knocking out ankyrin B, whereas the average length of the axons was significantly shortened in ankyrin B knockout neurons (Author response image 1B). These findings are consistent with the results presented in our recent paper (Lorenzo et al, *J. Cell Biol*, in press), which shows that ankyrin B is a major cargo adaptor for dynactin and promotes axonal transport. To quantify the tapering feature of dendrites, we measured the variance (standard deviation) of the dendrite diameters measured at five locations evenly spaced along each dendrite and obtained statistical average of the variance number across many dendrites. The diameter variance reflects the extent of the tapering with a smaller variance indicating a less tapered and more uniform neurite. The diameter variance appeared similar in dendrites of wild type and ankyrin B knockout neurons, suggesting that there was no substantial difference in the tapering of dendrites upon ankyrin B deletion (Author response image 1C). No significant changes were observed for the diameter variance of dendrites in the βII spectrin overexpressing neurons either (Author response image 1C). For comparison, we measured the variance of the axonal diameter and found it to be substantially smaller than those of the dendrites, confirming that the axons are indeed much less tapered (Author response image 1C).

In addition, we made an initial attempt to probe the axon-dendrite polarity in ankyrin B knockout neurons. Map2 and Tau are typically enriched in dendrites and axons, respectively. We therefore quantified the relative abundance of these proteins in dendrites and axons of wild type, and ankyrin B knockout neurons. The results shown in Author response image 1D illustrate that the relatively abundance of these proteins in dendrites vs. axons did not change substantially upon deletion of ankyrin B.

The above results suggest that dendrites largely retained its dendritic nature in ankyrin B knockout neurons. However, these simple quantifications do not exclude the possibility that other properties of dendrites may have changed upon ankyrin B deletion. More systematic characterizations of the properties of dendrites in ankyrin B knockout neurons await future studies. For this reason, we did not include these results in the revised manuscript.

**Author response image 1.** The dendrite morphology and axon-dendrite polarity of ankyrin B knockout neurons. A) Conventional images of Map2 and Tau in wild type and ankyrin B knockout (KO) neurons. B) The quantification of the average length of axons (left panel) and dendrites (right panel). C) Quantification of the diameter variance for dendrites from wild type, ankyrin B KO and βII spectrin overexpressing (OE) neurons, and the diameter variance for axons from ankyrin B knockout neurons. D) Quantification of Map2 and Tau fluorescence intensity in dendrites and axons from wild type and ankyrin B knockout neurons.

*4)* *Figure 1**: the presented neuron is rather atypical for a neuron 10 days in culture. They have very short dendrites, more reminiscent of immature minor neurites (please check Gomis-Rüth et al.,* *Nature Protocols, 2014). Please provide an example of more mature dendrites, and show the absence of the skeleton compared to the axon*.

As suggested, we have replaced Figure 1–figure supplement 1 with conventional and STORM images from a different DIV10 neuron that has longer and more elaborate dendrites. As shown by our STORM images (see revised Figure 1–figure supplement 1), the periodic structure is present in all the imaged axonal regions. We further imaged the dendrites of multiple DIV10 neurons, including this one shown in the revised Figure 1–figure supplement 1. Consistent with our observations for DIV2, DIV5 and DIV14 neurons shown in Figure 1–figure supplement 5, we didn’t observe the periodic structure in dendrites of DIV10 neurons. We have now included this additional quantification of DIV10 neurons in the revised Figure 1–figure supplement 5.

*5) In* *Figure 1A**, for images in A1, A2 and A3, how are the exposure times controlled for these images? Is it possible that the periodical distribution is already present in A2 but with less spectrin at each ring. As a result, when given the same exposure time as A1, less photo switching took place. In other words*, *is it possible that A2 is a partial pattern of A1?*

The images shown in the A1, A2 and A3 panels of Figure 1A were taken with the same exposure time. Indeed, the numbers of localizations in A2 and A3 are less (∼ 2 fold and ∼10 fold, respectively) than that in A1. However, since we projected the localization distribution into one dimension and performed the Fourier and autocorrelation analyses, if the highly regular periodic distribution were already present in A2 and A3, we would still have observed a pronounced peak in the Fourier analysis and a periodic function in the autocorrelation curve for A2 and A3. Indeed, when we randomly removed localizations from the A1 image to match up with the numbers of localizations in A2 and A3, the Fourier analyses still showed a much more pronounced peak than the corresponding ones for A2 and A3, and the autocorrelation analyses also showed a much clearer periodic function than the corresponding ones for A2 and A3 (see Author response image 2, below). Therefore, we do not think A2 is a simply partial pattern of the A1-like structure, but the periodicity of the distribution is indeed degraded towards the distal end of axons.

From Author response image 2A-C, we noticed that although the autocorrelation amplitude is not sensitive to the changes in the localization density, it did show a slight reduction as the number of localizations decreased to the A3 level. To quantify how much this effect contributes to the dependence of the autocorrelation amplitude on the distance to cell body shown in Figure 1E, we randomly removed localizations from the proximal axon regions to match the localization numbers measured in distal axons at various distances to the cell body, calculated the corresponding autocorrelation amplitudes for DIV2, 4 and 6 neurons, and included these results in the revised manuscript (see the new Figure 1–figure supplement 4). It is clear from these results that the change in the localization density along the axons does not contribute significantly to the change of the autocorrelation amplitude measured in Figure 1E.

**Author response image 2.** A) Top panel: STORM image of βII spectrin reproduced from Figure 1–A1. Middle panel: Fourier analysis of the localization distribution of this segment of the axon. Bottom panel: autocorrelation analysis of the same segment. B) Same as (A) except that some localizations are randomly removed from the A1 image so that the number of localizations matches that of the A2 image. C) Same as (A) except that some localizations are randomly removed from the A1 image so that the number of localizations matches that of the A3 image. D-E) Top panels: STORM images of βII spectrin reproduced from Figure 1–A2 and Figure 1–A3. Middle panels: Fourier analyses. Bottom panel: autocorrelation analyses.

*6) The earliest developmental stages that actin and spectrin rings can be detected are different. This is a bit troublesome to reconcile with the complete interdependence between actin and spectrin. The explanation given by the authors is that the actin rings might be lost during the fixation and staining procedures*. *Why not do these experiments with live neurites and mMaple labeled actin, which would bypass the fixation problem?*

We have actually tried to image actin in live neurons with the actin-mMaple3 fusion protein. However, it was difficult to observe the periodic actin structure at any stage of the neuron (immature or mature neurons) using this approach, even though the actin structure is clearly periodic in the axons of mature neurons as we showed in the images of fixed neurons with phalloidin labeling. This is due to a well-known problem: fusion constructs of actin and fluorescent proteins do not efficiently incorporate into the actin filaments and hence cannot give adequate quality super-resolution images of actin.

This fact has been pointed out in multiple previous papers (see, for example, Burkel et al., Cell Motil. Cytoskeleton, 64: 822-832, 2007; Kanchanawong et al., Nature, 468: 580-584, 2010). We have also tried an alternative approach of using LifeAct fused to mMaple3 to image actin in live neurons. LifeAct is a 17 amino acid peptide that binds to actin filaments. However, LifeAct presents its own difficulty because of the low binding affinity of LifeAct to actin (Kd = 2.2 μM; Riedl, et al., Nature Methods, 5: 605-607, 2008). Therefore, most of the LifeAct-mMaple3 were not associated with the periodic lattice structure, but were diffusing in the axons and gave a large background that obscured the periodic structure.

With future method development, it might be possible to directly observe the periodic actin distribution in the early developmental stages, but this would require a long-term effort and is beyond the scope of this paper. Given that the periodic structure of βII spectrin was rapidly and efficiently disrupted by actin-depolymerizing drugs, we think actin is involved in the lattice structure during early neuronal development. However, since periodic actin distribution was not directly observed during early developmental stages, we also clearly stated in the manuscript that it is formally possible that actin is not present in the periodic lattice structure during early developmental stages. However, we consider such a scenario to be less likely, as it is difficult to imagine how spectrin tetramers themselves could self-assemble into a periodic lattice structure without the help of actin to crosslink multiple spectrin tetramers. We carefully discussed these points in the manuscript in the Results section.

*7) It is not clear how ankyrin B prevents the localization of βII spectrin in dendrites. To prove that βII spectrin is responsible for the actin rings in the dendrites of ankyrin B knockout cells, the authors could knockdown βII spectrin in the ankyrin B mutant cells*.

We have now discussed a couple of possible mechanisms by which ankyrin B enriches βII spectrin in axons. See our response to comment #2 above as well as the text in the Discussion section of the revised manuscript.

As suggested here, we also tested whether βII spectrin knockdown affected the periodic lattice structure in the dendrites of ankyrin B knockout neurons. To study the dependence of the periodic lattice structure on βII spectrin, we monitored the periodic structure using a nonspectrin marker, adducin in this case. Indeed, adducin showed a periodic distribution in the dendrites of ankyrin B KO neurons. βII spectrin knockdown efficiently disrupted the periodic adducin structure in the dendrites of ankyrin B KO neurons. These new results suggest that βII spectrin is responsible for the formation of periodic lattice structure in the dendrites of ankyrin B knockout neurons. We have included these new results in the revised manuscript, in the Results section, and in Figure 7–figure supplement 2.

*8) It is not clear from the writing how microtubules contribute to the actin rings. The rationale of the microtubule experiments should be spelled out more clearly*.

As suggested, we have provided an explanation of our rationale to study the role of microtubules. Briefly, since the periodic lattice structure is specifically formed in axons during neuronal development, and microtubules are essential for the establishment of neuronal polarity and the formation of axons (Witte et al., J. Cell Biol, 180: 619-632, 2008), we wondered whether the formation of this periodic structure is dependent on microtubules.

Moreover, tubulin binds with ankyrin B, a molecule that also interacts with βII spectrin (Bennett et al, PNAS, 78: 7550-7554,1981). We thus tested whether microtubules play a role in the formation of the periodic structure. We have included these rationales in the revised manuscript, Results section. Our results indicate that the periodic lattice structure does depend on intact microtubules. Currently, we do not know the mechanism underlying the connection between microtubules and the period lattice structure yet.

*9) The invasion of the actin rings in the dendrites in the ankyrin B mutant cells provides the authors with the opportunity to understand the functional importance of these rings*. *Are there any morphological changes of the dendrites or defects in axon-dendrite polarity in these knockout neurons?*

Indeed, inducing the formation of the periodic lattice structure in dendrites by ankyrin B deletion provides an exciting opportunity to study the function of this lattice structure. We have followed the suggestion here and in comment #3 to study the dendrite morphology and axon-dendrite polarity in ankyrin B knockout neurons. We analyzed the lengths of the dendrites and the diameter uniformity (extent of tapering) of the dendrites, but did not observe any significant changes in these properties due to ankyrin B deletion. We also did preliminary analysis of the axon-dendrite polarity by measuring the abundance of MAP2 and Tau in dendrites and axons, and did not observe any significant change upon deletion of ankyrin B either. These results are shown in Author response image 1, in our response to comment #3 above.

These results suggest that dendrites largely retained its dendritic nature in ankyrin B knockout neurons. However, we note that these simple quantifications do not exclude the possibility that other properties of the dendrites may have changed upon ankyrin B deletion. For both dendrite morphology and axon-dendrite polarity, many more parameters could be measured.

There are also many other important properties in addition to morphology and polarity. A systematic characterization of the dendrite properties in ankyrin B knockout neurons would be a very interesting future direction but is beyond the scope of this manuscript, which is focused on the development mechanism of the periodic lattice structure in axons.
